# Hospital-acquired infections caused by enterococci: a systematic review and meta-analysis, WHO European Region, 1 January 2010 to 4 February 2020

**DOI:** 10.2807/1560-7917.ES.2021.26.45.2001628

**Published:** 2021-11-11

**Authors:** Simon Brinkwirth, Olaniyi Ayobami, Tim Eckmanns, Robby Markwart

**Affiliations:** 1 Unit 37: Nosocomial Infections, Surveillance of Antimicrobial Resistance and Consumption, Robert Koch Institute, Berlin, Germany; 2 Jena University Hospital, Institute of General Practice and Family Medicine, Jena, Germany

**Keywords:** *Enterococcus spp.*, vancomycin-resistant *Enterococcus spp.*, VRE, hospital-acquired infections, Hospital-acquired infections

## Abstract

**Background:**

Hospital-acquired infections (HAI) caused by *Enterococcus* spp., especially vancomycin-resistant *Enterococcus* *spp.* (VRE), are of rising concern.

**Aim:**

We summarised data on incidence, mortality and proportion of HAI caused by enterococci in the World Health Organization European Region.

**Methods:**

We searched Medline and Embase for articles published between 1 January 2010 and 4 February 2020. Random-effects meta-analyses were performed to obtain pooled estimates.

**Results:**

We included 75 studies. *Enterococcus* spp. and VRE accounted for 10.9% (95% confidence interval (CI): 8.7–13.4; range: 6.1–17.5) and 1.1% (95% CI: 0.21–2.7; range: 0.39–2.0) of all pathogens isolated from patients with HAI. Hospital wide, the pooled incidence of HAI caused by *Enterococcus* spp. ranged between 0.7 and 24.8 cases per 1,000 patients (pooled estimate: 6.9; 95% CI: 0.76–19.0). In intensive care units (ICU), pooled incidence of HAI caused by *Enterococcus* spp. and VRE was 9.6 (95% CI: 6.3–13.5; range: 0.39–36.0) and 2.6 (95% CI: 0.53–5.8; range: 0–9.7). Hospital wide, the pooled vancomycin resistance proportion among *Enterococcus* spp. HAI isolates was 7.3% (95% CI: 1.5–16.3; range: 2.6–11.5). In ICU, this proportion was 11.5% (95% CI: 4.7–20.1; range: 0–40.0). Among patients with hospital-acquired bloodstream infections with *Enterococcus* spp., pooled all-cause mortality was 21.9% (95% CI: 15.7–28.9; range: 14.3–32.3); whereas all-cause mortality attributable to VRE was 33.5% (95% CI: 13.0–57.3; range: 14.3–41.3).

**Conclusions:**

Infections caused by *Enterococcus* spp. are frequently identified among hospital patients and associated with high mortality.

## Introduction

*Enterococcus* spp. is a genus of Gram-positive, facultative anaerobic, catalase-negative bacteria that commonly inhabit the intestinal tracts of healthy humans and animals [1]. In addition to their role as commensals, enterococci are known for being associated with hospital-acquired infections. They can cause a wide range of infections, including infections of the urinary tract, bloodstream, and endocardium [2]. Enterococci, particularly *E. faecalis* and *E. faecium*, are among the most frequently isolated pathogens from patients with hospital-acquired infections (HAI) [1,3,4]. Hospital-acquired infections with enterococci are associated with considerable mortality [5-7], morbidity [8,9] and economic burden [10]. The clinical relevance of *Enterococcus* spp. is emphasised by their intrinsically low susceptibility to a wide range of antimicrobial drugs, including aminoglycosides, cephalosporins and sulphonamides and in the case of *E. faecium*, low-dose penicillin and ampicillin [11,12]. In view of the dwindling number of treatment options, vancomycin is commonly used to treat enterococcal infections, especially *E. faecium*. After the introduction of vancomycin in 1958 [13], a profound increase in prescriptions was recorded in the early 1980s [14]. Consequently, the first vancomycin resistance in clinical *Enterococcus* spp. isolates was observed in 1988 in London, United Kingdom [15]. Since then, vancomycin-resistant *Enterococcus* spp. (VRE) has spread and been detected in healthcare facilities across the world [16]. A rise of vancomycin resistance has been observed in clinical *Enterococcus* spp. isolates (especially in *E. faecium*) in many European countries in the last decade in particular [17,18]. A population-based study showed that there were ca 16,000 nosocomial VRE infections with 1,065 attributable deaths in the European Union/European Economic Area in 2015, nearly twice as many as reported in 2007 [19]. Aggregated data show that up to 55% of all HAI could be prevented by implementing multilevel infection prevention and control measures [20], potentially supporting a substantial reduction of the prevalence and mortality of enterococcal HAI. However, to our knowledge, no systematic review on the burden of HAI with *Enterococcus* spp., including vancomycin-resistant strains, in Europe has been published yet.

Systematic data on the epidemiology of enterococcal HAI are needed to fully estimate and understand the epidemiology of *Enterococcus* spp. infections. We therefore conducted a systematic review and meta-analysis to determine the prevalence, incidence and mortality as well as vancomycin resistance proportions of hospital-acquired *Enterococcus* spp. infections in the World Health Organization (WHO) European Region.

## Methods

We conducted this systematic review according to a protocol published a priori in the Prospective Register for Systematic Reviews (PROSPERO, 2020 CRD42020166863) and followed the reporting guidelines from the Preferred Reporting Items for Systematic Reviews and Meta-Analyses (PRISMA) statement [21].

### Study outcomes

The primary outcomes of this review are the prevalence, incidence and incidence density of hospital-acquired *Enterococcus* spp. / *E. faecium* and VRE / vancomycin-resistant *E. faecium* (VREF) infections among hospitalised patients and at the population level. Incidence density is defined as new cases per 1,000 patient hospitalisation days. The mortality of patients with HAI caused by *Enterococcus* spp. / *E. faecium* and VRE / VREF was additionally studied as a primary outcome. Secondary outcomes are (i) the proportion of vancomycin resistance among all *Enterococcus* spp. / *E. faecium* HAI isolates and; (ii) the proportion of HAI with *Enterococcus* spp. / *E. faecium* and VRE / VREF among all identified microorganisms from patients with HAI. In our review, cases of HAI caused by *Enterococcus* spp. / *E. faecium* include both vancomycin-resistant and sensitive strains.

### Search strategy, study selection criteria and data extraction

We searched Medline and Embase for epidemiological and surveillance studies reporting data on HAI. The search was carried out for studies published between 1 January 2010 and 4 February 2020 without any language restrictions. This timeframe was chosen because we aimed to summarise recent data on the epidemiology of hospital-acquired *Enterococcus* spp., especially given the rise of vancomycin resistance in Europe in the last decade. The detailed search strategy, including search strings, is provided in Supplementary Material. Title, abstract and full-text screening were independently performed by three authors (SB, OA, RM) using Covidence, a screening and data extraction tool recommended by the Cochrane Community [22]. All disagreements were discussed for consensus or resolved by a third reviewer.

The study selection criteria are presented in Box 1.

Box 1 **Study selection criteria**

Studies were included if they met all of the following criteria:

- The study provided data for at least one of the predefined primary outcomes for *Enterococcus* spp. and/or *E. faecium*. Studies were only included if they provided microbiological results where either the pathogen was identified or the culture was negative for more than 90% of all HAI episodes.
- The study was conducted in the WHO European Region.
- Data collection was completed before 2008 and the study was published after 2009.
- The hospital-acquired infections were defined according to appropriate definitions (e.g. US CDC/NHSN [99,100]).
- A largely unselected patient cohort was studied, i.e. not only high-risk patients such as low birthweight neonates or elderly patients, etc. or those with a specific underlying disease.
- The study was published in English, French, German or Spanish.
- Only studies that reported data for total HAI and HA-BSI were included.

Studies were excluded if:

- Data was provided for HAI outside of hospitals, such as nursing homes.
- Studies with any of the following study designs were excluded: literature reviews, intervention studies, case–control studies, outbreak studies and case series.

HAI: hospital-acquired infections; HA-BSI: hospital-acquired bloodstream infections; NHSN: National Healthcare Safety Network US CDC: United States Centers for Disease Control and Prevention; WHO: World Health Organization.

The data of all eligible studies were independently extracted by three authors (SB, OA and RM). All disagreements were resolved through discussion. The data extraction included the primary and secondary outcomes and the following study characteristics: authors, year of publication, study period, country, study design, setting (e.g. hospital, intensive care unit (ICU), etc.), age groups, patient inclusion criteria and the HAI infection type (i.e. total HAI and HA-BSI). We contacted study authors via email where details regarding outcomes and reporting were needed.

### Risk of bias assessment and statistical analysis

The risk of bias for individual studies was assessed by two authors (SB and RM) using the risk of bias tool developed by Hoy et al. [23]. For data analysis and presentation, studies were grouped into hospital-wide, ICU-based, neonatal ICU-based studies, and other hospital units/wards (e. g. internal medicine, surgical units, etc.) as well as by HAI types (i. e. total HAI and HA-BSI). All statistical analyses were performed using R version 3.6.1 and the R package meta version 4.9.7 (R Foundation, Vienna, Austria) [24]. Pooled estimates were calculated using random-effects models with a Tukey Double Arcsine transformation [25] of the raw proportions. The DerSimonian-Laird estimator was used to define τ^2^ (between-study variance). The I^2^ statistics quantified the statistical heterogeneity of the selected studies.

## Results

**Figure 1:**
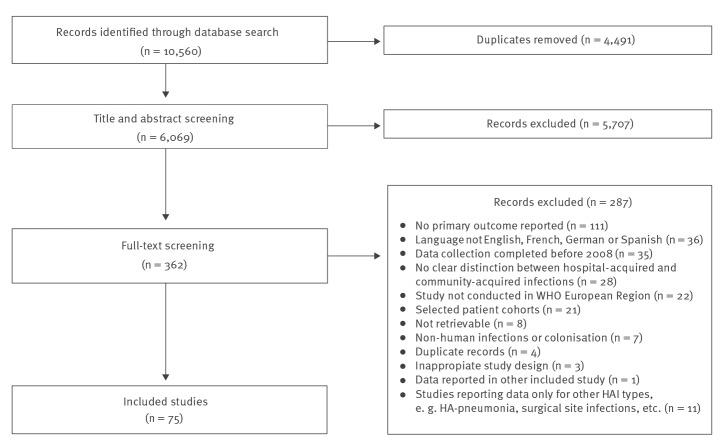
PRISMA flowchart of included studies on hospital-acquired infections caused by enterococci, WHO European Region, 1 January 2010−4 February 2020 (n = 75)

In total, we identified 6,069 unique records. After title and abstract screening, 362 studies were assessed in full-text review and 75 [6,7,26-98] met all inclusion criteria (Figure 1).

HA-pneumonia: hospital-acquired pneumonia; HAI: hospital-acquired infections; PRISMA: Preferred Reporting Items for Systematic Reviews and Meta-Analyses; WHO: World Health Organization.

### Study characteristics

The characteristics and individual study estimates are summarised in Supplementary Material, Table S1-S7. Among the 75 included studies, 28 [7,26-52] were conducted hospital wide, 34 [6,53-85] in ICU, nine [44,86-93] in neonatal ICU and five studies [94-98] were performed in other settings, such as internal medicine and surgical units. The studies were distributed across the WHO European Region (Supplementary Material, Figure S1. Geographical distribution of the included studies across the WHO European Region); studies from Turkey (n = 20), Italy (n = 10) and Poland (n = 9) were overrepresented. In total, nine studies were point prevalence studies, while the remaining 66 studies were incidence studies.

The results specifically for *E. faecium* and VREF are not presented in the main text of this study but are instead described in the Supplementary Material.

### Risk of bias assessment

The risk of bias for the representativeness of the studied hospital population was assessed as high in the majority of studies (69/75) (Supplementary Material, Table S8. Risk of bias assessment of included studies). Since these studies were single centre studies and/or included data from patients treated in academic medical centres, the representativeness of the included patients for the general hospital population in a given region or country was therefore unclear or low in these studies. Six studies [33,36,37,40,46,61] included nationally representative hospital populations. The risk of bias for the applied case definitions (i.e. hospital-acquired infections) was judged as low for most studies, since the majority of the studies (55/75) used HAI definitions based on the United States (US) Centers for Disease Control and Prevention criteria and the National Healthcare Safety Network criteria [99,100]. These validated definitions are widely used in the surveillance of HAI. More than half (46/75) of the studies did not report the used pathogen identification and/or antimicrobial susceptibility testing method and/or interpretation guideline (e.g. The European Committee on Antimicrobial Susceptibility Testing, Clinical and Laboratory Standards Institute). For this reason, the risk of bias with regards to the validity and reliability of the methodology used in these studies to identify enterococci and vancomycin-resistant strains was considered high (item 7, Supplementary Material, Table S8). In epidemiological surveys, HAI are typically defined as infections that occur 48 h after admission. That means that only patients with a hospital stay longer than 48 h in these studies are at risk of developing HAI and hence represent the appropriate denominator population for the parameters of interest (i.e. prevalence, incidence and mortality). Consequently, only studies including patients with a hospital stay longer than 48 h are judged as low risk of bias for item 10 (33/75 studies).

### Prevalence and incidence of hospital-acquired infections caused by *Enterococcus* spp. and vancomycin-resistant *Enterococcus* spp.

**Figure 2:**
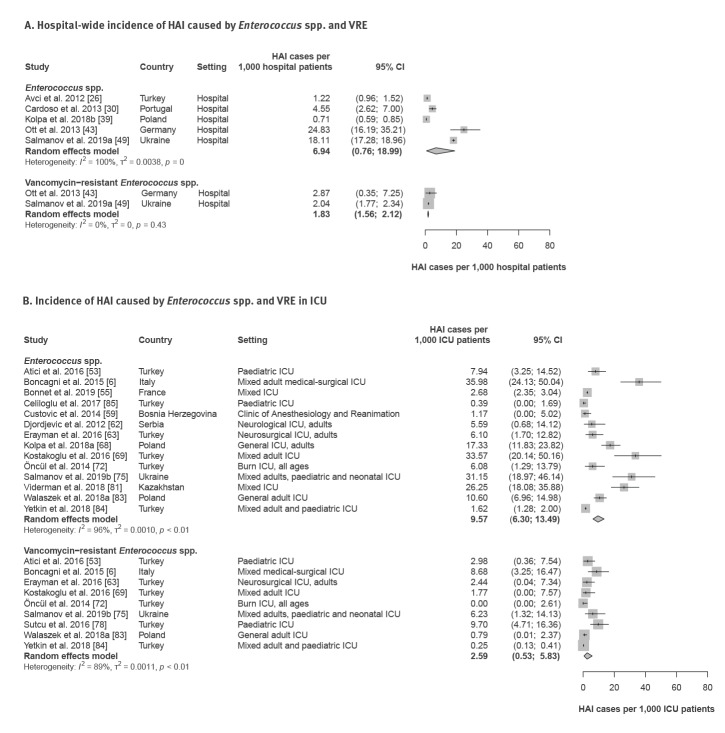
Incidence of hospital-acquired infections caused by *Enterococcus* spp. and vancomycin-resistant *Enterococcus* spp., WHO European Region, 1 January 2010–4 February 2020

CI: confidence interval; HAI: hospital-acquired infections; ICU: intensive care units; WHO: World Health Organization.

Five point prevalence studies [27,31,37,42,46] reported hospital-wide prevalence between 2.0 and 12.5 cases of *Enterococcus* spp. (including vancomycin-sensitive and -resistant strains) HAI per 1,000 hospital patients (pooled estimate: 4.6; 95% confidence interval (CI): 2.9–6.7) (Table). Similarly, based on five incidence studies, the pooled hospital-wide incidence of *Enterococcus* spp. HAI was 6.9 (95% CI: 0.76–19.0; range: 0.71–24.8) cases per 1,000 hospital patients (Figure 2A; Table). Two hospital-wide incidence studies reported 2.9 and 2.0 cases per 1,000 patients for HAI caused by VRE. For HA-BSI caused by *Enterococcus* spp., the hospital incidence ranged between 0.18 and 1.1 cases per 1,000 patients (pooled estimate: 0.62; 95% CI: 0.34–0.99, six studies) (Table).

**Table:** Summary of all primary outcomes on hospital-acquired infections caused by enterococci stratified by study setting, WHO European Region, 1 January 2010–4 February 2020

Study setting	Infection type	Pathogen	Number of studies	Pooled estimate (95% CI)	Inter-study heterogeneity (I^2^ statistics)	Range of individual study estimates
**Point prevalence (cases per 1,000 patients)**						
Hospital patients	All HAI	*Enterococcus spp.* ^a^	5 [29,33,39,44,48]	4.6 (2.96–.7)	48%	2.0–12.5
		VRE	NA			
	HA-BSI	*Enterococcus spp.* ^a^	3 [29,44,48]	0.63 (0.00–2.1)	27%	0–2.5
		VRE	NA			
ICU patients	All HAI	*Enterococcus spp.* ^a^	1 [81]	48.78 (22.91–83.1)	NA	
		VRE	NA			
	HA-BSI	*Enterococcus spp.* ^a^	3 [63,69,81]	5.5 (1.6–11.1)	15%	3.1–14.6
		VRE	1 [81]	9.8 (0.15–29.2)	NA	
**Incidence (new cases per 1,000 patients)**						
Hospital patients	All HAI	*Enterococcus spp.* ^a^	5 [28,32,41,45,51]	6.9 (0.76–19.0)	100%	0.71–24.8
		VRE	2 [45,51]	1.8 (1.6–2.1)	0%	2.0–2.9
	HA-BSI	*Enterococcus spp.* ^a^	6 [7,30,37,41,46,54]	0.62 (0.34–0.99)	97%	0.18–1.1
		VRE	1 [7]	0.37 (0.31–0.43)	NA	
ICU patients	All HAI	*Enterococcus spp.* ^a^	14 [6,55,57,61,64,65,70,71,77,83,85-87,102]	9.6 (6.3–13.5)	96%	0.39–36.0
		VRE	9 [6,53,63,69,72,75,78,83,84]	2.6 (0.53–5.8)	89%	0–9.7
	HA-BSI	*Enterococcus spp.* ^a^	12 [62,71,73,75-77,79,83,84,86,87,102]	6.1 (1.9–12.3)	97%	0–24.7
		VRE	8 [62,73,75,76,80,84,87,102]	0.06 (0.00–2.10)	79%	0–9.9
Neonatal ICU	All HAI	*Enterococcus spp.* ^a^	5 [89-93]	2.0 (0.05–5.7)	71%	0–15.9
		VRE	4 [89-92]	0 (0.00–0.32)	0%	0
	HA-BSI	*Enterococcus spp.* ^a^	6 [46,88-90,92,94]	2.3 (0.95–4.1)	61%	0–5.1
		VRE	4 [89-92]	0 (0.00–0.32)	0%	0
**Incidence density (cases per 1,000 patient days)**						
Hospital patients	All HAI	*Enterococcus spp.* ^a^	3 [28,41,43]	0.34 (0.08–0.78)	93%	0.14–0.92
		VRE	1 [43]	0.02	NA	
	HA-BSI	*Enterococcus spp.* ^a^	5 [31,38,40,41,49,54]	0.08 (0.05–0.12)	99%	0.03–0.14
		VRE	3 [31,40,49]	0.02 (0.00–0.06)	99%	0–0.12
ICU patients	All HAI	*Enterococcus spp.* ^a^	9 [6,55,64,70,71,77,85,87,102]	0.92 (0.41–1.60)	93%	0.05–2.57
		VRE	6 [6,55,71,77,85,102]	0.16 (0.03–0.37)	75%	0–0.62
	HA-BSI	*Enterococcus spp.* ^a^	8 [71,73,75,77,79,84,87,102]	0.61 (0.08–1.6)	96%	0–3.0
		VRE	5 [73,75,84,87,102]	0.01 (0.00–0.12)	70%	0–0.76
Neonatal ICU	All HAI	*Enterococcus spp.* ^a^	4 [89-92]	0.15 (0–0.54)	84%	0–1.5
		VRE	4 [89-92]	0 (0.00–0.01)	0%	0
	HA-BSI	*Enterococcus spp.* ^a^	3 [89,90,94]	0.11 (0.01–0.29)	84%	0.02–0.24
		VRE	3 [89-91]	0 (0.000.01)	0%	0
**All-cause mortality among patients with enterococcal HAI**						
Hospital patients	HA-BSI	*Enterococcus spp.* ^a^	5 [37,42,47,50,103]	21.9 (15.7–28.9)	85%	14.3–32.3
		VRE	2 [42,49]	33.5 (13.0–57.3)	45%	14.3–41.3
ICU patients	All HAI	*Enterococcus spp.* ^a^	1 [6]	31.0%	NA	
		VRE	2 [6,80]	33.0 (11.9–57.7)	0%	27.3–42.9
Neonatal ICU	HA-BSI	*Enterococcus spp.*	1 [94]	0%	NA	

CI: confidence interval; HAI: hospital-acquired infections; HA-BSI: hospital-acquired bloodstream infections; ICU: intensive care units; NA: not applicable; VRE: vancomycin-resistant *Enterococcus* spp.; WHO: World Health Organization.

^a^ Including vancomycin-sensitive and resistant strains.

The incidence of HAI caused by *Enterococcus* spp. and vancomycin-resistant *Enterococcus* spp. as cases per 1,000 patients. Cases of HAI caused by *Enterococcus* spp. include vancomycin-resistant and -sensitive strains. Pooled estimates were calculated using random-effects models with a Tukey Double Arcsine transformation of the raw proportions. The DerSimonian-Laird estimator was used to define τ^2^ (between-study variance).

Fourteen studies reported [6,53,55,59,62,63,68,69,72,75,81,82,84,85] data on the incidence of HAI caused by *Enterococcus* spp. in ICU. The individual study estimates ranged between 0.39 and 36.0 cases per 1,000 ICU patients (pooled estimate: 9.6; 95% CI: 6.3–13.5) (Figure 2B; Table). For HAI caused by VRE, the pooled estimate was 2.6 (95% CI: 0.5–5.8) cases per 1,000 ICU patients, with individual studies ranging from 0 to 9.7 cases per 1,000 patients. For HA-BSI, 12 studies reported ICU incidences between 0 and 24.7 *Enterococcus* spp. cases per 1,000 ICU patients (pooled estimate: 6.1; 95% CI: 1.9–12.3) (Table). Notably, two of eight studies identified cases of HA-BSI with VRE (range: 2.3–9.9). Data on the incidence and incidence density of HAI and HA-BSI caused by *Enterococcus* spp. in neonatal ICU are summarised in Table.

One study reported data on the population-based incidence or prevalence of enterococcal HAI. In this population-based study from Denmark [45], the incidence of monomicrobial enterococcal HA-BSI caused by *Enterococcus* spp. and VRE was 7.1 per 100,000 person-years and 0.1 per 100,000 person-years.

### Incidence density of hospital-acquired infections caused by *Enterococcus* spp. and vancomycin-resistant *Enterococcus* spp.

As shown by three studies [26,39,41], the hospital incidence density of HAI caused by *Enterococcus* spp. varied between 0.14 and 0.92 cases per 1,000 hospital patient days (pooled estimate: 0.34; 95% CI: 0.08–0.78) (Table). The ICU incidence density of hospital-acquired *Enterococcus* spp. ranged between 0.05 and 2.6 cases per 1,000 ICU patient days (pooled estimate: 0.92; 95% CI: 0.41–1.6, nine studies) (Table). For HAI caused by VRE, the pooled ICU incidence density was 0.16 (95% CI: 0.03–0.37), with an individual study range of 0 to 0.62 cases per 1,000 ICU patient days.

For HA-BSI caused by *Enterococcus* spp., five studies reported [29,36,38,39,52] incidence densities between 0.03 and 0.14 cases per 1,000 hospital patient days (pooled estimate: 0.08; 95% CI: 0.05–0.12) (Table). In ICU, the pooled incidence density for *Enterococcus* spp. HA-BSI was 0.61 (95% CI: 0.08–1.6) cases per 1,000 patient ICU days (eight studies, range: 0–3.0) (Table).

### Mortality

**Figure 3:**
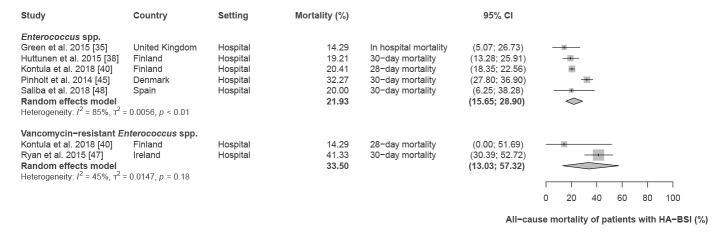
All-cause mortality of patients with hospital-acquired bloodstream infections caused by *Enterococcus* spp. and vancomycin-resistant *Enterococcus* spp., WHO European Region, 1 January 2010–4 February 2020

CI: confidence interval; HA-BSI: hospital-acquired bloodstream infections; HAI: hospital-acquired infections; ICU: intensive care units; WHO: World Health Organization.

The all-cause mortality recorded among patients with HA-BSI caused by *Enterococcus* spp. ranged between 14.3% and 32.3% (pooled estimate: 21.9%; 95% CI: 15.7–28.9, five studies) (Figure 3; Table). Based on two studies [40,47], the pooled all-cause mortality of patients with HA-BSI caused by VRE was 33.5% (95% CI: 13.0–57.3; range: 19.1–41.3). Importantly, Brady et al. 2017 [7] provided data on the attributable mortality of HA-BSI with enterococci. For *Enterococcus* spp. (including vancomycin-susceptible and -resistant strains) and VRE, this study reported an attributable mortality of 17.7% and 19.1%, respectively. This study also showed that the mortality of patients with HA-BSs caused by vancomycin-resistant *Enterococcus* spp. (19.1%) was similar to the mortality of HA-BSI patients with vancomycin-sensitive *Enterococcus* spp. (17.0%).

Pooled all-cause mortality (%) of patients with HA-BSI caused by *Enterococcus* spp. and vancomycin-resistant *Enterococcus* spp. Cases of HAI caused by *Enterococcus* spp. include vancomycin-resistant and -sensitive strains. Pooled estimates were calculated using random-effects models with a Tukey Double Arcsine transformation of the raw proportions. The DerSimonian-Laird estimator was used to define τ^2^ (between-study variance).

### Proportion of *Enterococcus* spp. and vancomycin-resistant *Enterococcus* spp. among all pathogens isolated from patient with HAI

**Figure 4:**
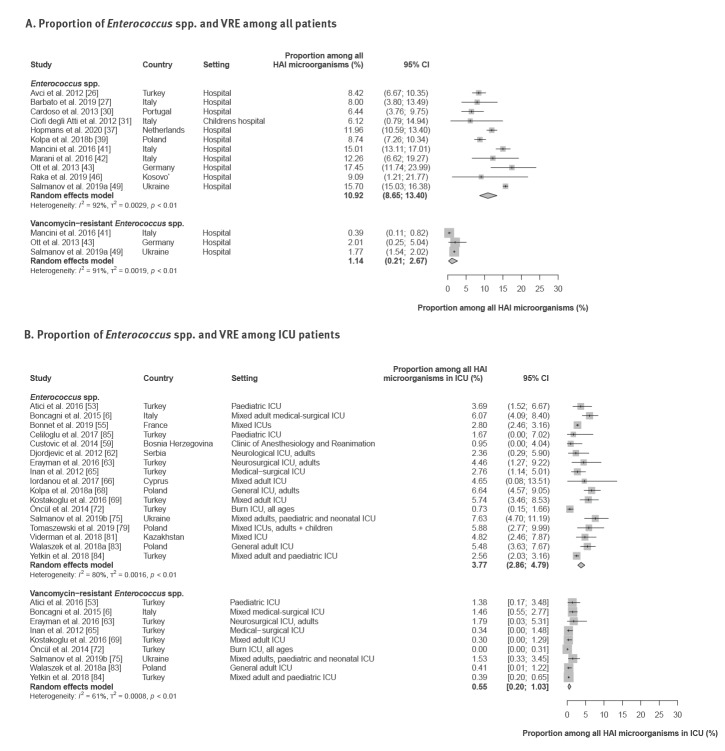
Proportion of *Enterococcus* spp. and vancomycin-resistant *Enterococcus* spp. among all microorganisms isolated from patients with hospital-acquired infections, WHO European Region, 1 January 2010–4 February 2020

CI: confidence interval; HAI: hospital-acquired infections; ICU: intensive care units; WHO: World Health Organization.

As reported by 11 hospital-wide studies, the proportion of *Enterococcus* spp. among all microorganisms isolated from HAI patients ranged between 6.1% and 17.5% (pooled estimate: 10.9%; 95% CI: 8.7–13.4) (Figure 4A; Supplementary Material, Table S6). Based on three studies [41,49,101], VRE isolates accounted for 0.39% to 2.0% (pooled estimate: 1.1%; 95% CI: 0.21–2.7) of all HAI pathogens.

Cases of HAI caused by *Enterococcus* spp. include vancomycin-resistant and -sensitive strains. Pooled estimates were calculated using random-effects models with a Tukey Double Arcsine transformation of the raw proportions. The DerSimonian-Laird estimator was used to define τ^2^ (between-study variance).

Compared with hospital-wide estimates, substantially lower *Enterococcus* spp. proportions were observed in HAI isolates from patients treated in ICU. Only 3.8% (95% CI: 2.9–4.8) of all isolated HAI microorganisms were identified as *Enterococcus* spp. (range: 0.73–7.6, 17 studies,) (Figure 4B; Supplementary Material, Table S6) in ICU. As reported by nine ICU studies [6,8,53,63,65,72,75,82,84], the proportion of VRE ranged between 0% and 1.8% (pooled estimate: 0.55%; 95% CI: 0.20–1.0).

In HA-BSI at the hospital level, the proportion of *Enterococcus* spp. among HA-BSI isolates ranged between 0% and 19.6% (pooled estimate: 9.2%; 95% CI: 6.9–11.7, 17 studies), while the proportion of VRE varied between 0% and 1.9% (Supplementary Material, Table S6). Compared with the hospital-wide estimates, similar proportions of *Enterococcus* spp. in HA-BSI isolates were observed in ICU (pooled estimate: 9.2%; 95% CI: 6.7–11.8; range: 0–28.6, 21 studies) (Supplementary Material, Table S6). In ICU, the proportion of VRE among all isolates from HA-BSI patients varied between 0% and 10.1% (pooled estimate: 1.3%; 95% CI: 0.16–3.2, 11 studies). In four of 11 studies, no VRE were found in isolates from ICU patients with HA-BSI.

### Proportion of vancomycin resistance among *Enterococcus* spp. isolates from patients with hospital-acquired infections

**Figure 5:**
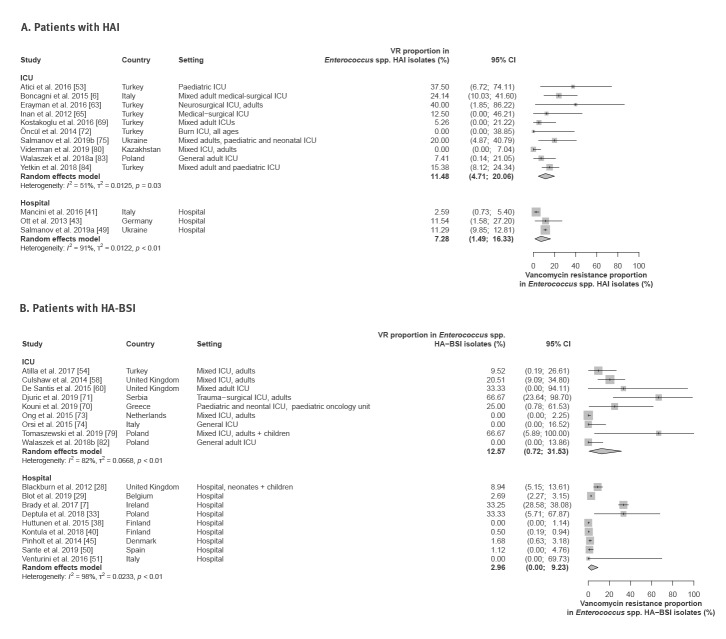
Proportion of vancomycin resistance among *Enterococcus* spp. isolates, hospital-wide and in intensive care units, WHO European Region, 1 January 2010–4 February 2020

CI: confidence interval; HAI: hospital-acquired infections; HA-BSI: hospital-acquired bloodstream infections; ICU: intensive care unit; WHO: World Health Organization.

Thirteen studies [6,41,43,49,53,63,65,69,72,75,80,82,84] provided data on vancomycin resistance proportion among all *Enterococcus* spp. isolates from patients with HAI in hospitals and ICUs. The VRE proportions ranged between 0% and 40% (pooled hospital-wide estimate: 7.3%; 95% CI: 1.5–16.3; pooled ICU estimate: 11.5%; 95% CI: 4.7–20.1) (Figure 5A; Supplementary Material, Table S7).

Pooled estimates were calculated using random-effects models with a Tukey Double Arcsine transformation of the raw proportions. The DerSimonian-Laird estimator was used to define τ^2^ (between-study variance).

In HA-BSI, a pooled vancomycin-resistant proportion of 3.0% (95% CI: 0–9.2) was observed hospital wide (Figure 5B; Supplementary Material, Table S7). Notably, while seven studies reported relatively low VRE proportions in HA-BSI (0–8.9%), two studies from Ireland [7] and Poland [33] reported high proportions of 33%. With the exception of three studies that found no vancomycin resistance, higher VRE proportions (range: 9.5–66.7, six studies) were found in HA-BSI *Enterococcus* spp. isolates from patients treated in ICU (Figure 5B; Supplementary Material, Table S7).

## Discussion

In view of limited treatment options, HAI with *Enterococcus* spp. are a serious health issue in the WHO European Region, particularly in light of increasing vancomycin resistance. This study is, to the best of our knowledge, the first systematic review to provide a comprehensive summary of data on the epidemiology of hospital-acquired infections caused by *Enterococcus* spp. and VRE in Europe.

The identified studies reported a hospital-wide point prevalence of HAI caused by *Enterococcus* spp. between 3.3 and 12.5 cases per 1,000 hospital patients, which is similar to Australia (8.0 cases per 1,000 patients) [102] and Latin America (4.0 cases per 1,000 patients) [103]. In contrast, lower prevalences of *Enterococcus* spp. HAI were observed in the US [104] and China [105], 1.9 and 1.3 cases per 1,000 hospital patients, respectively, which might be explained by generally lower hospital point prevalence of HAI in the US 3.2% [106] and in China 3.1% [107] compared with Europe 5.5% [4]. Another explanation might be broader screening practices and the implementation of contact precaution measures within the US healthcare system, particularly to control meticillin-resistant *Staphylococcus aureus* (MRSA) and VRE [108].

Our study emphasises the importance of *Enterococcus* spp. as a nosocomial pathogen, since it accounts for 6.1% to 17.5% of all pathogens isolated from patients with HAI. *Enterococcus* spp. usually remains among the top five most frequent nosocomial pathogens in Europe, despite the variation in species distribution across hospitals and regions [101,109-111]. In comparison, *Enterococcus* spp. is less frequently found in isolates from patients with HAI in the US [104] and China [105], 5% and 3.1% of all HAI pathogens, respectively. Our data show that VRE was found in 1.1% (range: 0.4–2.0) of all pathogens isolated from HAI patients, which is lower than the mean proportion of MRSA (ca 5%) observed in Europe [112]. However, in Germany [101] and Greece [109] VRE and MRSA are equally often found in HAI patients and in studies from Italy [110] and Ukraine [49], VRE is even more frequently isolated than MRSA, underlining the local heterogeneous distribution of nosocomial antibiotic-resistant pathogens. Interestingly, we found that *Enterococcus* spp. is less frequently isolated from HAI patients in ICU compared with patients treated hospital wide (10.9% vs 3.8%). However, the reasons for this observation are unclear.

Our study shows that the pooled vancomycin resistance proportions among HAI *Enterococcus* spp. were 7.3% hospital wide and 11.5% in isolates from patients in ICU, although individual study estimates varied somewhat. These pooled estimates are similar to the European Centre for Disease Prevention and Control data from the European Point Prevalence Survey [113,114]. In comparison to these European data, vancomycin resistance proportions are substantially lower in China [115,116] and Japan [117], where VRE proportions lower than 2% were observed. Interestingly, other countries in eastern Asia observed much higher VRE proportions, such as in South Korea (33.4%) [118] and Taiwan (40%) [119]. Compared with the European estimates, VRE proportions in the US are also generally higher (> 20%) [120,121], which might be explained by the widespread use of vancomycin in US hospitals, which increased by more than 30% between 2006 and 2012 [122].

For patients with HA-BSI caused by *Enterococcus* spp., all-cause mortality estimates ranged between 14.3% and 32.3% (pooled estimate: 21.1%). These are higher [35,38,40] or similar [7,48] to the all-cause mortality rates observed for *S. aureus* and generally higher than those reported for *E. coli* [7,35,38,40], which are other frequently encountered nosocomial pathogens. Substantial attention is paid to infection prevention and control (IPC) measures to address VRE, but our results show that enterococcal HAI as a whole are associated with a high incidence and mortality in Europe and should therefore receive more attention in IPC strategies.

An important observation of our study is that there is a large variation between individual study estimates of incidences/prevalences of HAI caused by *Enterococcus* spp. as well as for VRE proportions. This finding is similar to other systematic reviews around the world that also found large inter-study variations in the frequency of HAI [123-125]. Some of this heterogeneity might be explained by different methodological approaches, including different inclusion/exclusion criteria and microbiological sampling routines. In many published studies, data on the causative pathogen are not available for a substantial proportion of HAI episodes (> 40%) because of the lack of microbiological samples taken or incomplete data. This would ultimately lead to a substantial underestimation of the frequency of HAI caused by *Enterococcus* spp. To avoid this source of bias, we only included studies where pathogen identification results were reported for almost all HAI episodes. In addition to methodological differences, the large variation between individual study estimates also reflects true differences in the occurrence of nosocomial pathogens, including *Enterococcus* spp., between countries, regions and individual hospitals. For example, in a large multicentre study from Ukraine [49], *Escherichia coli*, *Staphylococcus aureus* and *Enterococcus* spp. were the predominant pathogens isolated from patients with HAI, while in a multicentre study from Greece, *Klebsiella* spp., *Pseudomonas aeruginosa* and *Acinetobacter* spp. were the most frequently identified nosocomial pathogens [126]. Furthermore, there is great variation in IPC policies and resources across Europe [127], which also explains the observed variations of HAI caused by *Enterococcus* spp. and VRE.

Since vancomycin resistance is predominantly found in *E. faecium* and less in *E. faecalis* and/or other enterococci species [7,18,75,118], vancomycin resistance proportions in *Enterococcus* spp. HAI isolates are also largely influenced by the proportion of *E. faecium* among all *Enterococcus* spp. isolates. Moreover, vancomycin resistance proportions in *E. faecium* differ across countries [18] and even within countries [17], which also explains the observed variation in VRE proportion described in our study. Moreover, nosocomial outbreaks and local spread of *E. faecium* genotypes associated with vancomycin resistance especially VanA and VanB in Europe [128] and increased virulence such as the *esp* and *hyl* genes can result in a higher VRE incidence in certain regions and hospitals. Another explanation for the observed inter-study variations in HAI caused by VRE are the profound differences in the consumption of glycopeptides/vancomycin, fluoroquinolones and third generation cephalosporins in Europe [129,130], whose usage is associated with VRE infections and colonisations in hospitals [131-135].

This systematic review is a comprehensive summary of recent data on the epidemiology of *Enterococcus* spp. and VRE in the WHO European Region, including 75 studies with data on over 8.5 million hospitalised patients with 154,000 HAI episodes. The majority of studies were based on routine HAI surveillance systems, including data from unselected patient cohorts. However, because of language restrictions in the literature selection, potentially relevant studies might have been excluded, for example from eastern European countries. Also, the majority of the included studies were conducted in academic medical centres and/or tertiary care hospitals and the representativeness of hospitalised patients and external validity of the study results might therefore be limited. Despite unclear representativeness of most studies, the overall quality of the studies and thus the quality of evidence was moderate to high. Another limitation is that many studies did not report vancomycin resistance profiles of *Enterococcus* spp. and data on the epidemiology of VRE are therefore limited. Importantly, since enterococci frequently colonise healthy people and are often detected in mixed infections, they may not be the causative microorganism in all HAI reported by the included studies. Especially in intra-abdominal, pelvic and soft tissue infections, the clinical relevance of *Enterococcus* spp. is debated [136]. Although the included studies were conducted in 21 different countries in the WHO European Region, the studies were not evenly distributed across Europe, which might lead to a geographical bias. For example, ICU-based studies were predominantly from studies in eastern and southern Europe and none was conducted in Scandinavia. Notably, studies from Turkey were overrepresented within the study set reporting VRE data. However, Turkish data did not systematically differ to data from other European countries. More nationally representative studies with complete microbiological and antimicrobial resistance profiles, including populations-based data, are needed in order to fully understand the epidemiology of HAI caused by *Enterococcus* spp. and VRE. In most analyses, a large statistical heterogeneity was observed (I^2^ > 80%) and the pooled estimates should be interpreted with caution. We therefore also provided the range of individual study estimates for all outcomes.

### Conclusions

Our data show that HAI caused by *Enterococcus* spp. and VRE are frequently identified among hospital patients and associated with high mortality in the WHO European Region. Continuous monitoring and the improved implementation of infection prevention and control programs as well as antibiotic stewardship measures are essential to reduce the burden of HAI caused by enterococci.

## *Note

This designation is without prejudice to positions on status, and is in line with UNSCR 1244/99 and the ICJ Opinion on the Kosovo declaration of independence.

## Supplementary Data

2212000 Supplement
