# Supplementary material for: Hospital-acquired infections caused by enterococci: a systematic review and meta-analysis, WHO European Region, 1 January 2010 to 4 February 2020
Source: Euro Surveill. 2021 Nov 11;26(45):2001628. doi: 10.2807/1560-7917.ES.2021.26.45.2001628 (PMC8646982; doi:10.2807/1560-7917.ES.2021.26.45.2001628)
Supplement: Supplement [file 20-01628_MARKWART_Supplementary_Material.pdf]

This supplementary material is hosted by *Eurosurveillance* as supporting information alongside the article *Hospital-acquired infections caused by enterococci: a systematic review and meta-analysis, WHO European Region, 1 January 2010 to 4 February 2020* on behalf of the authors, who remain responsible for the accuracy and appropriateness of the content. The same standards for ethics, copyright, attributions and permissions as for the article apply. Supplements are not edited by *Eurosurveillance* and the journal is not responsible for the maintenance of any links or email addresses provided therein.

Supplementary Material

Hospital-acquired infections caused by enterococci: a systematic review and meta-analysis, WHO European Region, 1 January 2010 to 4 February 2020

Contents

List of abbreviations 2

Search string 2

Supplementary Table S1. Characteristics of included studies 3

Supplementary Figure S1. Geographical distribution of the included studies across the WHO European Region ... 11

Supplementary Table S2. Point prevalence of HAIs due to *Enterococcus spp.* and vancomycin-resistant *Enterococcus spp.* (VRE) 12

Supplementary Table S3. Incidence of HAIs due to *Enterococcus spp.* and vancomycin-resistant *Enterococcus spp.* (VRE) 13

Supplementary Table S4. Incidence density of HAIs due to *Enterococcus spp.* and vancomycin-resistant *Enterococcus spp.* (VRE) 16

Supplementary Table S5. Mortality of patients with HAIs due to *Enterococcus spp.*, vancomycin-resistant *Enterococcus spp.* (VRE) or vancomycin-sensitive *Enterococcus spp.* (VSE) 19

Supplementary Table S6. Proportion of HAIs due to *Enterococcus spp.* and vancomycin-resistant *Enterococcus spp.* (VRE) among all microorganisms isolated from patients with HAIs 20

Supplementary Table S7. Vancomycin resistance proportions among *Enterococcus spp.* isolates from patients with HAIs 23

Supplementary Table S8. Risk of bias assessment of included studies 25

Results for hospital-acquired infections caused by *Enterococcus faecium* 28

Prevalence of hospital-acquired infections due to (vancomycin-resistant) Enterococcus faecium 28

Incidence of hospital-acquired infections with (vancomycin-resistant) Enterococcus faecium 29

Incidence density of hospital-acquired infections with (vancomycin-resistant) Enterococcus faecium 31

Mortality 33

The proportion of (vancomycin-resistant) Enterococcus faecium among all microorganisms causing HAIs 34

Vancomycin resistance proportions in Enterococcus faecium isolates from patients with HAIs 37

References of the Supplementary Material 38

List of abbreviations

AMR: Antimicrobial resistance; CDC: Centers for Disease Control and Prevention; CLABSI: Central line-associated bloodstream infection; CLSI = Clinical and Laboratory Standards Institute; CR-BSI: catheter-related/associated bloodstream infection; ECDC: European Centre for Disease Prevention and Control**;** EUCAST = European Committee on Antimicrobial Susceptibility Testing; HAIs: Hospital-Acquired Infections; HA-BSI: Hospital-acquired bloodstream infection; ICD-10: International Classification of Diseases, 10th revision; ICU: Intensive care unit; ISDA: Infectious Diseases Society of America; MO: Microorganism; NA: Not available; NHSN: National Healthcare Safety Network; NICU: Neonatal intensive care unit; primBSI: Primary bloodstream infection; secBSI: Secondary bloodstream infection; w/o: Without; VRE: Vancomycin-resistant *Enterococcus spp.*

Search string

(**'enterococcus faecium'** OR **'e. faecium'** OR **'enterococcus faecalis'** OR **'e. faecalis'** OR **'enterococcus spp*'** OR **'enterococcus species*'** OR **'enterococci'** OR **'vref'** OR **'vre'** OR **'vancomycin resistant enterococcus'** OR **'acinetobacter'** OR **'klebsiella'** OR **'escherichia'** OR **'streptococcus'** OR **'enterobacteriaceae'** OR **'pseudomonas'** OR **'staphylococcus'** OR **'gram-negative'** OR **'gram-positive'** OR **'e. coli'** OR **'k. pneumonia'** OR **'a. baumannii'** OR **'s. pneumoniae'** OR **'s. aureus'**)

AND

(**incidence** OR **prevalence** OR **'epidemiological data'** OR **'epidemiolog*'** OR **'frequenc*'** OR **population** OR **'population-based'** OR **surveillance** OR **'health survey'** OR **'mortalit*'** OR **'case fatalit*'**)

AND

(**'healthcare associated infection'** OR **'healthcare acquired infection'** OR **'hospital infection'** OR **'hospital acquired'** OR **'hospital associated'** OR **'nosocomial'** OR **'intensive care unit acquired infection'** OR **hai*** OR **'icu-a'** OR **'icu-acquired'**)

AND

(**'albania'** OR **'andorra'** OR **'armenia'** OR **'austria'** OR **'azerbaijan'** OR **'belarus'** OR **'belgium'** OR **'bosnia and herzegovina'** OR **'bulgaria'** OR **'croatia'** OR **'cyprus'** OR **'czech republic'** OR **'denmark'** OR **'estonia'** OR **'finland'** OR **'france'** OR **'georgia'** OR **'germany'** OR **'greece'** OR **'hungary'** OR **'iceland'** OR **'ireland'** OR **'israel'** OR **'italy'** OR **'kazakhstan'** OR **'kyrgyzstan'** OR **'latvia'** OR **'lithuania'** OR **'luxembourg'** OR **'malta'** OR **'monaco'** OR **'montenegro'** OR **'netherlands'** OR **'north macedonia'** OR **'macedonia'** OR **'norway'** OR **'poland'** OR **'portugal'** OR **'republic of moldova'** OR **'moldova'** OR **'romania'** OR **'russian federation'** OR **'russia'** OR **'san marino'** OR **'serbia'** OR **'slovakia'** OR **'slovenia'** OR **'spain'** OR **'sweden'** OR **'switzerland'** OR **'tajikistan'** OR **'turkey'** OR **'turkmenistan'** OR **'ukraine'** OR **'united kingdom'** OR **'scotland'** OR **'england'** OR **'wales'** OR **'northern ireland'** OR **'uzbekistan'** OR **'europe'** OR **'eu'** OR **'european union'** OR **'european economic area'**)

AND

[2010-2020]/py

AND

([article]/lim OR [article in press]/lim OR [data papers]/lim)

Supplementary Table S1. Characteristics of included studies

Study	Country	Study design; study period	Setting and patients	Definition of HAIs; Microbiology testing; AMR guidelines	Sample Size, No. of HAIs and identified microorganisms
Atici 2016 (1)	Turkey	Incidence, single centre; 2011-2014, 47 months	Paediatric ICU, training and research hospital (children)	CDC/NHSN (2); VITEK 2; CLSI	1007 patients, 224 HAIs, 227 MOs
Atilla 2017 (3)	Turkey	Incidence, single centre; 2008-2013, 79 months	Mixed ICU (medical, surgical, cardiovascular surgical, burn), education and training tertiary hospital (adults)	CDC/NHSN (4); Phoenix 100/BACTEC 9050; CLSI	17,553 patients, 166 CLBSIs, 172 MOs
Avci 2012 (5)	Turkey	Incidence, single centre; 2008-2009, 24 months	Training and research hospital (adults)	CDC/NHSN (2); NA; CLSI	60,585 patients, 825 HAIs, 879 MOs
Baier 2019 (6)	Germany	Incidence, single centre; 2016-2018, 17 months	Neonatal ICU, tertiary referral centre (neonates)	NA; VITEK 2; NA	671 patients, 23 HA-BSIs, 23 MOs
Barbato 2019 (7)	Italy	Point prevalence, single centre; 2018, 1 month	Teaching hospital (all age groups)	CDC/NHSN (4); NA; NA	799 patients, 38 HA-BSIs, 42 MOs
Blackburn 2012 (8)	United Kingdom	Incidence, multicentre; 2009-2010, 15 months	Hospitals from voluntary database LabBase2 (neonates and children)	CDC/NHSN (2); NA; NA	365,900 patients, 1734 HA-BSIs, 1734 MOs
Blot 2019 (9)	Belgium	Incidence, multicentre; 2000-2014, 180 months	Hospitals from national surveillance program (w/o specification of included age groups)	CDC/NHSN (2); NA; NA	NA, 59,941 HA-BSIs, 66,610 MOs
Bolat 2012 (10)	Turkey	Incidence, single centre, 2009-2011, 24 months	Neonatal ICU, education and teaching hospital (neonates)	CDC/NHSN (2); BACTEC 9240; CLSI	1395 patients, 227 HAIs, 278 MOs
Boncagni 2015 (11)	Italy	Incidence, single centre, 2009-2012, 48 months	Mixed medical-surgical ICU, tertiary teaching hospital (adults)	CDC/NHSN (4); NA; NA	806 patients, 289 HAIs, 478 MOs
Bonnet 2019	France	Incidence, multicentre,	29 mixed ICUs, surveillance and	CDC/NHSN (4);	87,931 patients,

(12)		2000-2013,<br>156 months	control network (all age groups)	NA; EUCAST	10,001 HAIs,<br>8428 MOs
Brady 2017 (13)	Ireland	Incidence, multicentre; 2007-2013,<br>84 months	6 hospitals, teaching and non-teaching (all age groups)	NA; NA; NA	343,189 patients,<br>NA,<br>NA
Candevir 2011 (14)	Turkey	Incidence, single centre; 2006-2009,<br>48 months	4 ICUs (traumatic, general medical and surgical, general, medical neurosurgical, and paediatric medical and surgical), teaching hospital (children and adults)	CDC/NHSN (2); VITEK 2; CLSI	6834 patients,<br>283 CA-HAI,<br>698 MOs
Cardoso 2013 (15)	Portugal	Incidence, single centre; 2008-2009,<br>12 months	University tertiary care hospital (adults)	CDC/NHSN (2); VITEK 2; NA	3733 patients,<br>317 HAIs,<br>264 MOs
Celiloğlu 2017 (16)	Turkey	Incidence, single centre; 2012-2016,<br>60 months	Level III paediatric ICU, training and research hospital (children)	CDC/NHSN (4); NA	2545 patients,<br>60 HAIs,<br>60 MOs
Çevik 2013 (17)	Turkey	Incidence, single centre; 2007-2009,<br>34 months	Medical ICU, teaching hospital (adults)	CDC/NHSN (4); VITEK 2; NA	1650 patients,<br>NA,<br>134 MOs
Ciofi degli Atti 2012 (18)	Italy	Point prevalence, single centre; 2007-2010,<br>8 weeks	Paediatric hospital, tertiary care teaching and research hospital (children)	CDC/NHSN (4); NA; NA	1506 patients,<br>102 HAIs,<br>49 MOs
Crivaro 2015 (19)	Italy	Incidence, single centre; 2006-2010,<br>60 months	Level III neonatal ICU, university hospital (neonates)	CDC/NHSN (4); NA; NA	1699 patients,<br>48 CLABSIs,<br>48 MOs
Culshaw 2014 (20)	United Kingdom	Incidence, single centre; 2009-2013,<br>59 months	Tertiary mixed medical and surgical ICU, teaching hospital (adults)	CDC/NHSN (4); NA	NA,<br>31 CLABSIs,<br>32 MOs
Cura 2016 (21)	Turkey	Incidence, single centre; 2012-2013,<br>12 months	Neonatal ICU, tertiary teaching hospital (neonates)	CDC/NHSN (4); BACTEC; CLSI	377 patients,<br>68 HAI,<br>51 MOs
Custovic 2014 (22)	Bosnia Herzegovina	Incidence, single centre; 2013,<br>12 months	ICU, university clinic of anaesthesiology and reanimation (w/o specification of included age groups)	CDC/NHSN; VITEK 2; NA	855 patients,<br>105 HAIs,<br>105 MOs
De Angelis 2018	Italy	Incidence,	Teaching hospital (all	Own definition;	681,082 patients,

(23)		single centre; 2007-2015, 108 months	age groups)	VITEK 2; EUCAST	NA, NA
De Santis 2015 (24)	United Kingdom	Incidence, single centre; 2012-2013, 6 months	General medical-surgical ICU, university hospital (adults)	Own definition; NA; NA	1318 patients, 37 HA-BSI, 37 MOs
Deptuła 2017 (25)	Poland	Point prevalence, multicentre; 2012-2014, 4 months	ICUs from voluntarily participating hospitals in ECDC PPS (adults)	ECDC (26); NA; NA	945 patients, 61 HA-BSIs, 61 MOs
Deptuła 2018 (27)	Poland	Point prevalence, multicentre; 2012-2015, 5 months	Voluntarily participating hospitals from ECDC PPS (all age groups)	ECDC (26); NA; NA	71,360 patients, 161 CR-BSIs, 161 MOs
Djordjevic 2012 (28)	Serbia	Incidence, single centre; 2009-2010, 12 months	Neurological ICU, university hospital (adults)	CDC/NHSN (2); NA; NA	537 patients, 101 HAI, 127 MOs
Djordjevic 2015 (29)	Serbia	Incidence, single centre; 2012, 12 months	Neonatal ICU, university hospital (neonates)	CDC/NHSN (2); NA; NA	381 patients, 74 HAI, 56 MOs
Djuric 2019 (30)	Serbia	Incidence, single centre; 2014-2016, 18 months	2 trauma-surgical ICUs, teaching hospital (adults)	CDC/NHSN (4); VITEK 2; CLSI	406 patients, 62 HA-BSI, 69 MOs
Erayman 2016 (31)	Turkey	Incidence, single centre; 2010-2014, 48 months	Neurosurgery ICU, teaching hospital (adults)	CDC/NHSN; NA; NA	820 patients, 118 HAI, 112 MOs
Erdem 2015 (32)	Turkey	Incidence, single centre; NA, 12 months	Research and training hospital (w/o specification of included age groups)	NA; NA; NA	4675 patients, 59 CLABSIs, 60 MOs
Gecgel 2016 (33)	Turkey	Incidence, single centre; 2011-2015, 57 months	Clinical departments and ICU of cardiology and cardiovascular surgery, tertiary training and research hospital (w/o specification of included age groups)	CDC/NHSN; BACTEC; NA	27,886 patients, 273 HA-BSIs, 273 MOs
Geffers 2011 (34)	Germany	Incidence, multicentre; 2005-2009, 60 months	ICUs from voluntarily national database (w/o specification of included age groups)	CDC/NHSN; NA; NA	1,651,941 patients, 5067 CR-BSIs, 5067 MOs
Green 2015	United Kingdom	Incidence,	Hospitals from	ICD-10; NA; NA	62,676 patients,

(35)		multicentre; 2009, 3 months	voluntary database LabBase2 (children)		214 HA-BSIs 214 MOs
Gubbels 2017 (36)	Denmark	Incidence, multicentre; 2010-2014, 60 months	Hospitals from voluntarily national database (w/o specification of included age groups)	CDC/NHSN (4); NA	NA, 13,704 HA-BSIs, 13,704 MOs
Guembe 2017 (37)	Spain	Incidence, multicentre; 2015-2016, 12 months	14 internal medicine departments, teaching and non-teaching hospitals (adults)	Own definition; NA; NA	42,577 patients, 70 CR-BSIs, 72 MOs
Hopmans 2020 (38)	Netherlands	Point prevalence, multicentre; 2014-2016, 36 months	Voluntarily participating teaching and non-teaching hospitals (all age groups)	ECDC (26); NA; NA	49,734 patients, 2687 HAIs, 2057 MOs
Huttunen 2015 (39)	Finland	Incidence, single centre; 2005-2010, 72 months	Tertiary hospital (all age groups)	CDC/NHSN (2); BACTEC 9240/ BacT/ALERT 3D; CLSI/ EUCAST	NA, 2175 HA-BSIs, 1941 MOs
Inan 2012 (40)	Turkey	Incidence, single centre; 2004-2010 (data for 2010 were extracted, 12 months)	Medical-surgical ICU, teaching hospital (w/o specification of included age groups)	CDC/NHSN (2, 4); NA; CLSI	1007 patients, NA, 290 MOs
Iordanou 2017 (41)	Cyprus	Incidence, single centre; 2015, 12 months	Mixed ICU, secondary general referral hospital (adults)	CDC/NHSN; Phoenix+Vitek2; NA	198 patients, 43 DA-HAIs, 43 MOs
Kepenekli 2015 (42)	Turkey	Point prevalence, multicentre; 2012, 1 day	50 paediatric ICUs, teaching and research hospitals (children)	CDC/NHSN (4); NA; NA	327 patients, 28 HA-BSIs, 32 MOs
Kołpa 2018a (43)	Poland	Incidence, single centre; 2007-2016, 120 months	Generals ICU, non-teaching secondary care hospital (adults)	ECDC (44); NA; NA	1847 patients, 510 HAIs, 482 MOs
Kołpa 2018b (45)	Poland	Incidence, single centre; 20012-2016, 60 months	Non-teaching secondary care hospital (all age groups)	ECDC (26); NA; NA	159,028 patients, 2184 HAIs, 1293 MOs
Kołpa 2019 (46)	Poland	Incidence, single centre; 2003-2017, 180 months	Surgical unit including ICU, non-teaching secondary care hospital (w/o specification of	ECDC (26, 44); Phoenix; NA	10,332 patients, 476 HAIs, 404 MOs

			included age groups)		
Kontula 2018 (47)	Finland	Incidence, multicentre; 1999-2014, 192 months	18 hospitals with 4 tertiary, 9 secondary and 5 other acute care hospitals (all age groups)	CDC/NHSN (2); NA; NA	NA, 17,767 HA-BSI, 17,767 MOs
Kostakoğlu 2016 (48)	Turkey	Incidence, single centre; 2013, 12 months	4 ICUs (Anaesthesia and Reanimation, Surgical, Medical, and Neurology), teaching hospital (adults)	CDC/NHSN (4); Phoenix; NA	566 patients 309 HAI, 331 MOs
Kouni 2019 (49)	Greece	Incidence, multicentre; 2016-2017, 9 months	All state hospitals including NICUs, PICUs and ONC (neonates+children)	CDC/NHSN; NA; NA	NA, 111 CLABSIs, 123 MOs
Kuzdan 2014 (50)	Turkey	Incidence, single centre; 2008-2010, (data for 2009+2010 were extracted, 24 months)	Paediatric ward with ICU, teaching hospital (children)	CDC/NHSN (2); VITEK 2; CLSI	1194 patients, 241 HAIs, 241 MOs
Mancini 2016 (51)	Italy	Incidence, single centre; 2008-2010, 40 months	Tertiary care hospital (all age groups)	CDC/NHSN; NA; NA	NA, NA, 1286 MOs
Marani 2016 (52)	Italy	Point prevalence surveys, single centre; 2011-2015	Teaching hospital without inclusion of ICU (all age groups)	CDC/NHSN (2); NA; NA	2840 patients, 110 HAIs, 106 MOs
Öncül 2014 (53)	Turkey	Incidence, single centre; 2001-2012, 144 months	Burn ICU, teaching hospital (all age groups)	CDC/NHSN (2); API Rapid ID 32 Staph; CLSI	658 patients, 602 HAIs, 547 MOs
Ong 2015 (54)	Netherlands	Incidence, multicentre; 2011-2013, 27 months	2 mixed ICUs, tertiary hospitals (adults)	CDC/NHSN (2, 4); NA; NA	3080 patients, 266 HA-BSIs, 266 MOs
Orsi 2015 (55)	Italy	Incidence, single centre; 2000-2007 and 2010-2012 (data for 2010-2012 were extracted, 36 months)	General ICU, teaching hospital (adults)	CDC/NHSN (2); VITEK; CLSI/ EUCAST	1165 patients, 101 HA-BSIs, 115 MOs
Ott 2013 (56)	Germany	Incidence, single centre; 2010, 2 months	University hospital (adults)	CDC/NHSN (4); NA; NA	1047 patients, 124 HAIs, 149 MOs

Pérerz Lopéz 2013 (57)	United Kingdom	Incidence, single centre; 2001-2009, 108 months	Tertiary referral university hospital with a NICU (children and neonates)	Own definition; BacTec; NA	49,179 patients, 478 HAIs, 524 MOs
Pinholt 2014 (58)	Denmark	Incidence, multicentre; 2006-2009, 48 months	Teaching and non-teaching hospitals (adults)	CDC/NHSN (2); VITEK 2; EUCAST	NA, NA, NA
Raka 2019 (59)	Kosovo	Point-prevalence, multicentre; in 2016	1 tertiary care hospital, 6 secondary care hospitals (all age groups)	ECDC (26); NA; NA	915 patients, 45 HAIs, 33 MOs
Ryan 2015 (60)	Ireland	Incidence, single centre; 2009-2012, 42 months	University hospital (adults)	ECDC (61); VITEK 2; EUCAST	NA, NA, NA
Sadowska-Krawczenko 2012 (62)	Poland	Incidence, single centre; 2005-2010, 72 months (data for 2009-2010 were extracted, 24 months)	Level III neonatal ICU, university hospital (neonates)	CDC/NHSN (2); BacT/Alert; NA	853 patients, 77 HAIs, 77 MOs
Saliba 2018 (63)	Spain	Incidence, single centre; 2004-2014, 132 months	Tertiary care teaching hospital without inclusion of ICUs (adults)	IDSA (64); BACTEC; NA	NA, 546 CR-BSIs, 560 MOs
Salmanov 2019a (65)	Ukraine	Incidence, multicentre; 2014-2016, 36 months	17 hospitals, general, paediatric and women´s (all age groups)	CDC/NHSN (4); VITEK 2; CLSI	97,340 patients, 10,986 HAIs, 11,231 MOs
Salmanov 2019b (66)	Ukraine	Incidence, multicentre; 2012-2014, 36 months	Medical and surgical ICUs in 4 city hospitals (all age groups)	CDC/NHSN (4); VITEK 2; CLSI	642 patients, 148 HAIs, 262 MOs
Sante 2019 (67)	Spain	Incidence, single centre; 2009-2014, 72 months	Tertiary care and teaching hospital (all age groups)	CDC/NHSN (4); NA; NA	147,931 patients, 429 HA-secBSIs, 519 MOs
Schwab 2018 (68)	Germany	Incidence, multicentre; 2006-2015, 120 months	937 mixed ICUs from national surveillance (all age groups)	CDC/NHSN (4); NA; NA	4,556,360 patients, 12,745 HA-primBSIs, 12,745 MOs
Süner 2015 (69)	Turkey	Incidence, single centre; 2011, 6 months	Central ICU, university hospital (adults)	CDC/NHSN (4); BacT/Alert + VITEK 2; NA	407 patients, 64 HA-BSIs, 64 MOs
Sutcu 2016 (70)	Turkey	Incidence, single centre;	Paediatric ICU, tertiary care and	CDC/NHSN (4); VITEK 2; CLSI	1134 patients,

		2010-2014, 60 months	teaching hospital (children)		NA, NA
Tomaszewski 2019 (71)	Poland	Point-prevalence, multicentre; 2014, 1 day	28 ICUs, without specification of included hospitals, (children+ adults)	CDC/NHSN (4); NA; EUCAST	205 patients, 134 HAIs, 170 MO
Tsitsopoulos 2016 (72)	Greece	Incidence, single centre; 2003-2012, 120 months	Neurosurgery unit including intermediate care, teaching hospital (w/o specification of included age groups)	CDC/NHSN (4); VITEK 2; CLSI	5933 patients, 326 HA-BSIs, 378 MO
Venturini 2016 (73)	Italy	Incidence, single centre; 2014-2015, 6 months	Tertiary care and teaching hospital (children)	CDC/NHSN; NA; NA	NA, 29 CLABSIs, 27 MO
Verstraete 2014 (74)	Belgium	Incidence, single centre; 1992-2011, 240 months (data for 2002-2011 were extracted, 120 months)	Neonatal ICU, tertiary referral centre and teaching hospital (neonates)	CDC/NHSN (4); BacT/Alert; NA	5329 patients, 418 HA-BSIs, 418 MO
Viderman 2018 (75)	Kazakhstan	Incidence, single centre; 2014-2016, 23 months	Mixed ICU, national research centre for oncology and transplantation (adults)	NA; NA; CLSI	1257 patients, 249 HAIs, 249 MO
Viderman 2019 (76)	Kazakhstan	Incidence, single centre; 2015, 12 months	Mixed ICU, national research centre for oncology and transplantation (adults)	NA; VITEK; CLSI	546 patients, 137 HAIs, 137 MO
Virano 2015 (77)	Italy	Incidence, single centre; 2010-2011, 12 months	Paediatric referral and teaching hospital (neonates+ children)	CDC/NHSN (2, 4); BacT/Alert; NA	14,342 patients, 91 HA-BSIs, 98 MO
Walaszek 2018a (78)	Poland	Incidence, multicentre; 2013-2015, 36 months	7 general ICUs, non-teaching hospitals (adults)	ECDC (26, 44); NA; EUCAST	2547 patients, 575 HAIs, 493 MO
Walaszek 2018b (79)	Poland	Incidence, multicentre; 2013-2015, 36 months	7 general ICUs, non-teaching hospitals (adults)	ECDC (26, 44); NA; EUCAST	2547 patients, 97 HA-BSIs, 78 MO
Yalaz 2012 (80)	Turkey	Incidence, single centre; 2008-2010, 36 months	Level III neonatal ICU, university hospital (neonates)	CDC/NHSN (2); NA; NA	600 patients, 9 CR-BSIs, 9 MO
Yetkin 2018	Turkey	Incidence, single centre;	10 ICUs, tertiary care and teaching hospital	CDC/NHSN (2, 4);	48,263 patients,

(81)		2007-2015,<br>105 months	(all age groups)	VITEK 2; CLSI	4272 HAIs,<br>3044 MOs

Supplementary Figure S1. Geographical distribution of the included studies across the WHO European Region

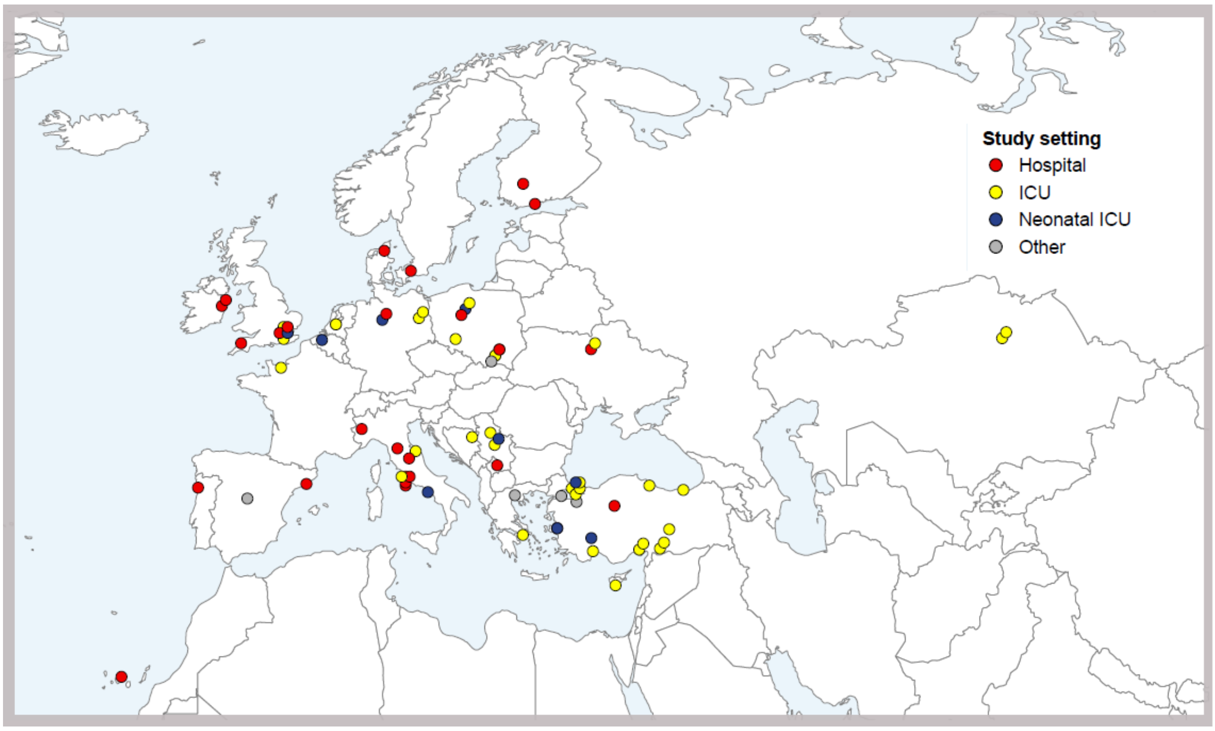

Supplementary Table S2. Point prevalence of HAIs due to *Enterococcus spp.* and vancomycin-resistant *Enterococcus spp.* (VRE)

**Study**		**Hospital wide (cases per 1000 hospital patients)**		**ICU (cases per 1000 ICU patients)**		**NICU (cases per 1000 NICU patients)**	
		Enterococcus spp.	**VRE**	***Enterococcus spp.***	**VRE**	***Enterococcus spp.***	**VRE**
**All hospital-acquired infections**							
Barbato 2015	Italy, hospital wide	12.52	-	-	-	-	-
Ciofi degli Atti 2012	Italy, hospital wide	1.99	-	-	-	-	-
Hopmans 2018	Netherlands, hospital wide	4.95	-	-	-	-	-
Marani 2016	Italy, hospital wide (w/o ICU)	4.58	-	-	-	-	-
Raka 2019	Kosovo, hospital wide	3.28	-	-	-	-	-
Tomaszewski 2019	Poland, mixed ICUs, adults + children	-	-	48.78	-	-	-
**Hospital-acquired bloodstream infections**							
Barbato 2015	Italy, hospital wide	2.5	-	-	-	-	-
Marani 2016	Italy, hospital wide (w/o ICU)	0.70	-	-	-	-	-
Raka 2019	Kosovo, hospital wide	0	-	-	-	-	-
Deptuła 2017	Poland, mixed ICUs, adults	-	-	5.29	-	-	-
Kepenekli 2015	Turkey, paediatric ICU	-	-	3.06	-	-	-
Tomaszewski 2019	Poland, mixed ICUs, adults + children	-	-	14.63	9.76	-	-
**Catheter-associated bloodstream infections**							
Deptuła 2018	Poland, hospital wide	0.13	0.04	-	-	-	-

Supplementary Table S3. Incidence of HAIs due to *Enterococcus spp.* and vancomycin-resistant *Enterococcus spp.* (VRE)

Study		Hospital wide (cases per 1000 patients)		ICU (cases per 1000 patients)		NICU (cases per 1000 patients)		Other wards (cases per 1000 patients)	
		Enterococcus spp.	**VRE**	***Enterococcus spp.***	**VRE**	***Enterococcus spp.***	**VRE**	***Enterococcus spp.***	**VRE**
**All hospital-acquired infections**									
Avci 2012	Turkey, hospital wide	1.22	-	-	-	-	-	-	-
Cardoso 2013	Portugal, hospital wide	4.55	-	-	-	-	-	-	-
Kołpa 2018b	Poland, hospital wide	0.71	-	-	-	-	-	-	-
Ott 2013	Germany, hospital wide	24.83	2.87	-	-	-	-	-	-
Salmanov 2019a	Ukraine, hospital wide	18.11	2.04	-	-	-	-	-	-
Atici 2016	Turkey, Paediatric ICU	-	-	7.94	2.98	-	-	-	-
Boncagni 2015	Italy, mixed medical-surgical ICUs, adults	-	-	35.98	8.68	-	-	-	-
Bonnet 2019	France, mixed ICUs, all ages	-	-	2.68	-	-	-	-	-
Celiloğlu 2017	Turkey, paediatric ICU	-	-	0.39	-	-	-	-	-
Custovic 2014	Bosnia Herzegovina, clinic of anaesthesiol ogy and reanimation	-	-	1.17	-	-	-	-	-
Djordjevic 2012	Serbia, neurological ICU, adults	-	-	5.59	-	-	-	-	-
Erayman 2016	Turkey, neurosurgical ICU, adults	-	-	6.10	2.44		-	-	-
Iordanou 2017	Cyprus, mixed ICU, adults	-	-	10.10	-	-	-	-	-
Kołpa 2018a	Poland, general ICU, adults	-	-	17.33	-	-	-	-	-
Kostakoğlu 2016	Turkey, mixed ICUs, adults	-	-	33.57	1.77	-	-	-	-
Öncül 2014	Turkey, burn ICU, all ages	-	-	6.08	0	-	-	-	-
Salmanov 2019b	Ukraine, mixed adults, paediatric and neonatal ICUs	-	-	31.15	6.23	-	-	-	-
Sutcu 2016	Turkey, paediatric ICU	-	-	-	9.70	-	-	-	-
Viderman 2018	Kazakkhstan, mixed ICU	-	-	26.25	-	-	-	-	-
Viderman 2019	Kazakkhstan, mixed ICU, adults	-	-	51.28	-	-	-	-	-
Walaszek 2018a	Poland, general adult ICUs	-	-	10.60	0.79	-	-	-	-
Yetkin 2018	Turkey, mixed adult and paediatric ICUs	-	-	1.62	0.25	-	-	-	-
Bolat 2012	Turkey, neonatal ICU	-	-	-	-	2.15	0	-	-
Crivaro 2015	Italy, neonatal ICU	-	-	-	-	0.59	0	-	-
Cura 2016	Turkey,	-	-	-	-	15.92	0	-	-

	neonatal ICU								
Djordjevic 2015	Serbia, neonatal ICU	-	-	-	-	0	0	-	-
Sadowska-Krawczenko 2012	Poland, neonatal ICU	-	-	-	-	1.17	-	-	-
Kołpa 2019	Poland, surgical unit	-	-	-	-	-	-	0	0
**Hospital-acquired bloodstream infections**									
Blackburn 2012	United Kingdom, hospital wide	0.59	-	-	-	-	-	-	-
Brady 2017	Ireland, hospital wide	1.10	0.37	-	-	-	-	-	-
Green 2015	United Kingdom, hospital wide	0.67	-	-	-	-	-	-	-
Kołpa 2018b	Poland, hospital wide	0.18	-	-	-	-	-	-	-
Pérerz Lopéz 2013	United Kingdom, hospital wide	0.79	-	-	-	-	-	-	-
Virano 2015	Italy, hospital wide	0.63	-	-	-	-	-	-	-
Celiloglu 2017	Turkey, paediatric ICU	-	-	0	0	-	-	-	-
De Santis 2015	United Kingdom, mixed ICU, adults	-	-	5.31	2.28	-	-	-	-
Djuric 2019	Serbia, trauma-surgical ICUs, adults	-	-	14.78	9.85	-	-	-	-
Kostakoğlu 2016	Turkey, mixed ICUs, adults	-	-	12.37	-	-	-	-	-
Öncül 2014	Turkey, burn ICU, all ages	-	-	0	0	-	-	-	-
Ong 2015	Netherlands, mixed ICUs, adults	-	-	24.68	0	-	-	-	-
Orsi 2015	Italy, general ICU, adults	-	-	8.58	0	-	-	-	-
Salmanov 2019b	Ukraine, mixed adults, paediatric and neonatal ICUs	-	-	7.79	-	-	-	-	-
Süner 2015	Turkey, central ICU, adults	-	-	14.74	-	-	-	-	-
Sutcu 2016	Turkey, paediatric ICU	-	-	6.17	-	-	-	-	-
Viderman 2018	Kazakkhstan, mixed ICU	-	-	9.55	-	-	-	-	-
Walaszek 2018b	Poland, general adult ICUs	-	-	4.71	0	-	-	-	-
Yetkin 2018	Turkey, mixed adult and paediatric ICUs	-	-	0.58	-	-	-	-	-
Baier 2019	Germany, neonatal ICU	-	-	-	-	2.98	-	-	-
Bolat 2012	Turkey, neonatal ICU	-	-	-	-	1.43	0	-	-
Crivaro 2015	Italy, neonatal ICU	-	-	-	-	0.59	0	-	-
Cura 2016	Turkey, neonatal ICU	-	-	-		-	0	-	-
Djordjevic 2015	Serbia, neonatal ICU	-	-	-	-	0	0	-	-
Pérerz Lopéz 2013	United Kingdom, neonatal ICU	-	-	-	-	3.65	-	-	-
Verstraete 2014	Belgium, neonatal ICU	-	-	-	-	5.07	-	-	-
Gecgel	Turkey,	-	-	-	-	-	-	1.18	-

2016	clinical departments and ICUs of cardiology and cardiovascular surgery								
Kołpa 2019	Poland, surgical unit	-	-	-	-	-	-	0	0
Tsitsopoulos 2016	Greece, neurosurgical unit	-	-	-	-	-	-	7.92	0
**Hospital-acquired primary bloodstream infections**									
Schwab 2018	Germany, mixed ICUs, all ages	-	-	0.46	-	-	-	-	-
Walaszek 2018a	Poland, general adult ICUs	-	-	2.75	-	-	-	-	-
Walaszek 2018b	Poland, general adult ICUs	-	-	3.53	0	-	-	-	-
**Hospital-acquired secondary bloodstream infections**									
Sante 2019	Spain, hospital wide	0.60	0.007	-	-	-	-	-	-
Walaszek 2018b	Poland, general adult ICUs	-	-	1.18	0	-	-	-	-
**Catheter-associated bloodstream infections**									
Çevik 2013	Turkey, medical ICU, adults	-	-	-	8.48	-	-	-	-
Djuric 2019	Serbia, trauma-surgical ICUs, adults	-	-	0	0	-	-	-	-
Geffers 2011	Germany, mixed ICUs	-	-	0.57	-	-	-	-	-
Yalaz 2012	Turkey, neonatal ICU	-	-	-	-	1.67	-	-	-
Guembe 2017	Spain, internal medicine, adults	-	-	-	-	-	-	0.09	-
**Central line-associated bloodstream infections**									
Erdem 2015	Turkey, hospital wide	0.86	-	-	-	-	-	-	-
Atilla 2017	Turkey, mixed ICUs, adults	-	-	1.20	0.11	-	-	-	-
Crivaro 2015	Italy, neonatal ICU	-	-	-	-	0.59	0	-	-
**Device-associated infections**									
Inan 2012	Turkey, medical-surgical ICU	-	-	7.94	0.99	-	-	-	-
Iordanou 2017	Cyprus, mixed ICU, adults	-	-	10.10	-	-	-	-	-

Supplementary Table S4. Incidence density of HAIs due to *Enterococcus spp.* and vancomycin-resistant *Enterococcus spp.* (VRE)

Study		Hospital wide		ICU		NICU		Other wards	
		Enterococcus spp.	**VRE**	**Enterococcus spp.**	**VRE**	***Enterococcus spp.***	**VRE**	***Enterococcus spp.***	**VRE**
**All hospital-acquired infections**									
Avci 2012	Turkey, hospital wide	0.18 cases per 1000 patient days	-	-	-	-	-	-	-
Kołpa 2018b	Poland, hospital wide	0.14 cases per 1000 patients	-	-	-	-	-	-	-
Mancini 2016	Italy, hospital wide	0.92 cases per 1000 patient days	0.02 cases per 1000 patient days	-	-	-	-	-	-
Atici 2016	Turkey, Paediatric ICU	-	-	0.74 cases per 1000 patient days	0.28 cases per 1000 patient days	-	-	-	-
Boncagni 2015	Italy, mixed medical-surgical ICUs, adults	-	-	2.57 cases per 1000 patient days	0.62 cases per 1000 patient days	-	-	-	-
Celiloğlu 2017	Turkey, paediatric ICU	-	-	0.05 cases per 1000 patients	-	-	-	-	-
Djordjevic 2012	Serbia, neurological ICU, adults	-	-	0.46 cases per 1000 patient days	-	-	-	-	-
Kołpa 2018a	Poland, general ICU, adults	-	-	1.82 cases per 1000 patients	-	-	-	-	-
Kostakoğlu 2016	Turkey, mixed ICUs, adults	-	-	1.56 cases per 1000 patient days	0.08 cases per 1000 patient days	-	-	-	-
Öncül 2014	Turkey, burn ICU, all ages	-	-	0.15 cases per 1000 patient days	0 cases per 1000 patient days	-	-	-	-
Salmanov 2019b	Ukraine, mixed adults, paediatric and neonatal ICUs	-	-	1.23 cases per 1000 patient days	0.25 cases per 1000 patient days	-	-	-	-
Walaszek 2018a	Poland, general adult ICUs	-	-	1.35 cases per 1000 patient days	0.10 cases per 1000 patient days	-	-	-	-
Bolat 2012	Turkey, neonatal ICU	-	-	-	-	0.14 cases per 1000 patient days	0 cases per 1000 patient days	-	-
Crivaro 2015	Italy, neonatal ICU	-	-	-	-	0.02 cases per 1000 patient days	0 cases per 1000 patient days	-	-
Cura 2016	Turkey, neonatal ICU	-	-	-	-	1.50 cases per 1000 patients	0 cases per 1000 patient days	-	-
Djordjevic 2015	Serbia, neonatal ICU	-	-	-	-	0 cases per 1000 patient days	0 cases per 1000 patient days	-	-
**Hospital-acquired bloodstream infections**									
Blot 2019	Belgium, hospital wide	0.07 cases per 1000 patient days	0.002 cases per 1000 patient days	-	-	-	-	-	-

Gubbels 2017	Denmark, hospital wide	0.13 cases per 1000 patient days	-	-	-	-	-	-	-
Huttunen 2015	Finland, hospital wide	0.07 cases per 1000 patient days	0 cases per 1000 patient days	-	-	-	-	-	-
Kołpa 2018b	Poland, hospital wide	0.03 cases per 1000 patients	-	-	-	-	-	-	-
Ryan 2015	Ireland, hospital wide	-	0.12 cases per 1000 patient days	-	-	-	-	-	-
Virano 2015	Italy, hospital wide	0.14 cases per 1000 patients	-	-	-	-	-	-	-
Celiloğlu 2017	Turkey, paediatric ICU	-	-	0 cases per 1000 patients days	0 cases per 1000 patients days	-	-	-	-
Djuric 2019	Serbia, trauma-surgical ICUs, adults	-	-	1.14 cases per 1000 patient days	0.76 cases per 1000 patient days	-	-	--	-
Kostakoğlu 2016	Turkey, mixed ICUs, adults	-	-	0.58 cases per 1000 patient days	-	-	-	-	-
Öncül 2014	Turkey, burn ICU, all ages	-	-	0 cases per 1000 patient days	0 cases per 1000 patient days	-	-	-	-
Ong 2015	Netherlands, mixed ICUs, adults	-	-	3.00 cases per 1000 patient days	0 cases per 1000 patient days	-	-	-	-
Salmanov 2019b	Ukraine, mixed adults, paediatric and neonatal ICUs	-	-	0.31 cases per 1000 patient days	-	-	-	-	-
Süner 2015	Turkey, central ICU, adults	-	-	1.71 cases per 1000 patients	-	-	-	-	-
Walaszek 2018b	Poland, general adult ICUs	-	-	0.60 cases per 1000 patient days	0 cases per 1000 patient days	-	-	-	-
Bolat 2012	Turkey, neonatal ICU	-	-	-	-	0.09 cases per 1000 patient days	0 cases per 1000 patient days	-	-
Crivaro 2015	Italy, neonatal ICU	-	-	-	-	0.02 cases per 1000 patient days	0 cases per 1000 patient days	-	-
Cura 2016	Turkey, neonatal ICU	-	-	-	-	-	0 cases per 1000 patient days	-	-
Verstraete 2014	Belgium, neonatal ICU	-	-	-	-	0.24 cases per 1000 patients	-	-	-
**Hospital-acquired primary bloodstream infections**									
Schwab 2018	Germany, mixed ICUs, all ages	-	-	0.12 cases per 1000 patients	-	-	-	-	-
Walaszek 2018a	Poland, general adult ICUs	-	-	0.35 cases per 10000 patient days	-	-	-	-	-
Walaszek 2018b	Poland, general adult ICUs	-	-	0.45 cases per 1000 patient days	0 cases per 1000 patient days	-	-	-	-
**Hospital-acquired secondary bloodstream infections**									
Sante 2019	Spain, hospital wide	0.09 cases per 1000	0.001 cases	-	-	-	-	-	-

		patient days	per 1000 patient days						
Walaszek 2018b	Poland, general adult ICUs	-	-	0.15 cases per 1000 patient days	0 cases per 1000 patient days	-	-	-	-
**Catheter-associated bloodstream infections**									
Saliba 2018	Poland, hospital wide w/o ICU	0.01 cases per 1000 patients	-	-	-	-	-		
Çevik 2013	Turkey, medical ICU, adults	-	-	-	0.71 cases per 1000 patients	-	-	-	-
Djuric 2019	Serbia, trauma-surgical ICUs, adults	-	-	0 cases per 1000 patients	0 cases per 1000 patients	-	-	-	-
Geffers 2011	Germany, mixed ICUs	-	-	0.16 cases per 1000 patients	-	-	-	-	-
Yalaz 2012	Turkey, neonatal ICU	-	-	-	-	0.10 cases per 1000 patients	-	-	-
**Central line-associated bloodstream infections**									
Erdem 2015	Turkey, hospital wide	0.14 cases per 1000 patients / 0.25 cases per 1000 catheter days	-	-	-	-	-	-	-
Venturini 2016	Italy, chospital wide	0.26 cases per 1000 catheter days	0 cases per 1000 catheter days	-	-	-	-	-	-
Atilla 2017	Turkey, mixed ICUs, adults	-	-	0.22 cases per 1000 patient days / 0.54 cases per 1000 catheter days	0.02 cases per 1000 patient days / 0.05 cases per 1000 catheter days	-	-	-	-
Culshaw 2014	Italy, mixed ICU, adults	-	-	0.08 cases per 1000 catheter days	0.03 cases per 1000 catheter days	-	-	-	-
Kouni 2019	Greece, paediatric and neonatal ICUs, paediatric oncology unit	-	-	0.10 cases per 1000 patient days / 0.32 cases per 1000 catheter days	0.02 cases per 1000 patient days / 0.08 cases per 1000 catheter days	-	-	-	-
Crivaro 2015	Italy, neonatal ICU	-	-	-	-	0.02 cases per 1000 patient days / 0.17 cases per 1000 catheter days	0 cases per 1000 patient days / cathete r days	-	-
**Device-associated infections**									
Inan 2012	Turkey, medical-surgical ICU	-	-	1.10 cases per 1000 patient days	0.14 cases per 1000 patient days	-	-	-	-

Supplementary Table S5. Mortality of patients with HAIs due to *Enterococcus spp.*, vancomycin-resistant *Enterococcus spp.* (VRE) or vancomycin-sensitive *Enterococcus spp.* (VSE)

Study		Hospital wide			ICU			NICU
		Enterococcus spp.	**VRE**	**VSE**	***Enterococcus spp.***	**VRE**	**VSE**	***Enterococcus spp.***
**All hospital-acquired infections**								
Boncagni 2015	Italy, mixed medical-surgical ICUs, adults	-	-	-	31.03% (crude mortality)	42.86% (crude mortality)	27.27% (crude mortality)	-
Sutcu 2016	Turkey, paediatric ICU	-	-	-	-	27.27% (crude mortality)	-	-
**Hospital-acquired bloodstream infections**								
Brady 2017	Ireland, hospital wide	17.68% (attributable mortality)	19.05% (attributable mortality)	17% (attributable mortality)	-	-	-	-
Green 2015	United Kingdom, hospital wide	14.29% (in hospital mortality))	-	-	-	-	-	-
Huttunen 2015	Finland, hospital wide	19.21% (30-day crude mortality) / 9.27% (7-day crude mortality)	-	-	-	-	-	-
Kontula 2018	Finland, hospital wide	20.41% (30-day crude mortality) / 9.43% (7-day crude mortality)	14.29% (30-day crude mortality) / 14.29% (7-day crude mortality)	20.44% (30-day crude mortality) / 9.40% (7-day crude mortality)	-	-	-	-
Pinholt 2014	Denmark, hospital wide	32.27% (30-day monomicrobial mortality)	-	-	-	-	-	-
Ryan 2015	Ireland, hospital wide	-	41.33% (30-day crude mortality) / 22.67% (7-day crude mortality)	-	-	-	-	-
Ong 2015	Netherlands, mixed ICUs, adults	-	-	-	47.37% (attributable ICU-mortality)	-	-	-
Orsi 2015	Italy, general ICU, adults	-	-	-	50% (microorganism associated mortality)	-	-	-
Tsitsopoulos 2016	Greece, neurosurgical unit	-	-	-	-	-	-	53.19% (28-day crude mortality)
Verstraete 2014	Belgium, neonatal ICU	-	-	-	-	-	-	0% (associated mortality)
**Hospital-acquired primary bloodstream infections**								
Schwab 2018	Germany, mixed ICUs, all ages	-	-	-	21.71% (attributable ICU-mortality)	-	-	-
**Catheter-associated bloodstream infections**								
Saliba 2018	Spain, hospital wide w/o ICU	20% (30-day crude mortality)	-	-	-	-	-	-

Supplementary Table S6. Proportion of HAIs due to *Enterococcus spp.* and vancomycin-resistant *Enterococcus spp.* (VRE) among all microorganisms isolated from patients with HAIs

Study		Hospital wide		ICU		NICU		Other wards		Comment
		Enterococcus spp.	**VRE**	***Enterococcus spp.***	**VRE**	***Enterococcus spp.***	**VRE**	***Enterococcus spp.***	**VRE**	
**All hospital-acquired infections**										
Avci 2012	Turkey, hospital wide	8.42%	-	-	-	-	-	-	-	-
Barbato 2015	Italy, hospital wide	8%	-	-	-	-	-	-	-	-
Cardoso 2013	Portugal, hospital wide	6.44%	-	-	-	-	-	-	-	-
Ciofi degli Atti 2012	Italy, hospital wide	6.12%	-	-	-	-	-	-	-	-
Hopmans 2020	Netherlands, hospital wide	11.96%	-	-	-	-	-	-	-	-
Kołpa 2018b	Poland, hospital wide	8.74%	-	-	-	-	-	-	-	-
Mancini 2016	Italy, hospital wide	15.01%	0.39%	-	-	-	-	-	-	-
Marani 2016	Italy, hospital wide w/o ICU	12.26%	-	-	-	-	-	-	-	-
Ott 2013	Germany, hospital wide	17.45%	2.02%	-	-	-	-	-	-	-
Raka 2019	Kosovo, hospital wide	9.09%	-	-	-	-	-	-	-	-
Salmanov 2019a	Ukraine, hospital wide	15.70%	1.77%	-	-	-	-	-	-	-
Atici 2016	Turkey, Paediatric ICU	-	-	3.69%	1.38%	-	-	-	-	-
Boncagni 2015	Italy, mixed medical-surgical ICUs, adults	-	-	6.07%	1.46%	-	-	-	-	-
Bonnet 2019	France, mixed ICUs, all ages	-	-	2.8%	-	-	-	-	-	-
Celiloğlu 2017	Turkey, paediatric ICU	-	-	1.67%	-	-	-	-	-	-
Custovic 2014	Bosnia Herzegovina, clinic of anaesthesiology and reanimation	-	-	0.95%	-	-	-	-	-	-
Djordjevic 2012	Serbia, neurological ICU, adults	-	-	2.36%	-	-	-	-	-	-
Erayman 2016	Turkey, neurosurgical ICU, adults	-	-	4.46%	1.79%	-	-	-	-	-
Inan 2012	Turkey, medical-surgical ICU	-	-	2.76%	0.34%	-	-	-	-	Device-associated infections
Iordanou 2017	Cyprus, mixed ICU, adults	-	-	4.65%	-	-	-	-	-	Device-associated infections
Kołpa 2018a	Poland, general ICU, adults	-	-	6.64%	-	-	-	-	-	-
Kostakoğlu 2016	Turkey, mixed ICUs, adults	-	-	5.74%	0.3%	-	-	-	-	-
Öncül 2014	Turkey, burn ICU, all ages	-	-	0.73%	0%	-	-	-	-	-
Salmanov 2019b	Ukraine, mixed adults, paediatric and neonatal ICUs	-	-	7.63%	1.53%	-	-	-	-	-
Tomaszewski 2019	Poland, mixed ICUs, adults + children	-	-	5.88%	-	-	-	-	-	-
Viderman	Kazakhstan,	-	-	4.82%	-	-	-	-	-	-

2018	mixed ICU									
Viderman 2019	Kazakkhstan, mixed ICU, adults	-	-	20.44%	-	-	-	-	-	-
Walaszek 2018a	Poland, general adult ICUs	-	-	5.48%	0.41%	-	-	-	-	-
Yetkin 2018	Turkey, mixed adult and paediatric ICUs	-	-	2.56%	0.39%	-	-	-	-	-
Bolat 2012	Turkey, neonatal ICU	-	-	-	-	1.08%	0%	-	-	-
Cura 2016	Turkey, neonatal ICU	-	-	-	-	11.76%	0%	-	-	-
Djordjevic 2015	Serbia, neonatal ICU	-	-	-	-	0%	0%	-	-	-
Sadowska-Krawczenko 2012	Poland, neonatal ICU	-	-	-	-	1.3%	-	-	-	-
Kołpa 2019	Poland, - surgical unit	-	-	-	-	-	-	-	0%	-
**Hospital-acquired bloodstream infections**										
Barbato 2015	Italy, hospital wide	4.76%	-	-	-	-	-	-	-	-
Blackburn 2012	United Kingdom, hospital wide	12.51%	-	-	-	-	-	-	-	-
Blot 2019	Belgium, hospital wide	7.8%	0.21%	-	-	-	-	-	-	-
Deptuła 2018	Poland, hospital wide	5.59%	1.86%	-	-	-	-	-	-	Catheter-associated bloodstream infections
Erdem 2015	Turkey, hospital wide	6.67%	-	-	-	-	-	-	-	Central line-associated bloodstream infections
Green 2015	United Kingdom, hospital wide	19.63%	-	-	-	-	-	-	-	-
Gubbels 2017	Denmark, hospital wide	18.1%	-	-	-	-	-	-	-	-
Huttunen 2015	Finland, hospital wide	7.78%	0%	-	-	-	-	-	-	-
Kołpa 2018b	Poland, hospital wide	9.33%	-	-	-	-	-	-	-	-
Kontula 2018	Finland, hospital wide	7.94%	0.04%	-	-	-	-	-	-	-
Marani 2016	Italy, hospital wide (w/o ICU)	9.52%	-	-	-	-	-			-
Pérerz Lopéz 2013	United Kingdom, hospital wide	7.44%	-	-	-	-	-	-	-	-
Raka 2019	Kosovo, hospital wide	0%	-	-	-	-	-	-	-	-
Saliba 2018	Spain, hospital wide w/o ICU	4.46%	-	-	-	-	-	-	-	Catheter-associated bloodstream infections
Sante 2019	Spain, hospital wide	17.15%	0.19%	-	-	-	-	-	-	Secondary bloodstream infections
Venturini 2016	Italy, hospital wide	7.41%	0%	-	-	-	-	-	-	Central line-associated bloodstream infections
Virano 2015	Italy, hospital wide	9.18%	-	-	-	-	-	-	-	-
Atilla 2017	Turkey, mixed ICUs, adults	-	-	12.21%	1.16%	-	-	-	-	Central line-associated bloodstream infections
Celiloğlu 2017	Turkey, paediatric ICU	-	-	0%	-	-	-	-	-	-
Çevik 2013	Turkey, medical ICU, adults	-	-	-	10.07%	-	-	-	-	Catheter-associated bloodstream infections

Culshaw 2014	Italy, mixed ICU, adults	-	-	7.26% (all HA-BSI); 9.38% (central line-associated BSI)	1.49% (all HA-BSI) 3.13%; (central line-associated BSI)	-	-	-	-	-
De Santis 2015	United Kingdom, mixed adult ICU	-	-	8.11%	2.7%	-	-	-	-	-
Deptuła 2017	Poland, mixed ICUs, adults	-	-	8.2%	-	-	-	-	-	-
Djuric 2019	Serbia, trauma-surgical ICUs, adults	-	-	8.7% (all HA-BSI); 0% (catheter-associated BSI)	5.8% (all HA-BSI); 0% (catheter-associated BSI)	-	-	-	-	-
Geffers 2011	Germany, mixed ICUs	-	-	18.49%	-	-	-	-	-	Catheter-associated bloodstream infections
Kepenekli 2015	Turkey, paediatric ICU	-	-	3.13%	-	-	-	-	-	-
Kostakoğlu 2016	Turkey, mixed ICUs, adults	-	-	4.7%	-	-	-	-	-	-
Kouni 2019	Greece, paediatric and neonatal ICUs, paediatric oncology unit	-	-	6.5%	1.63%	-	-	-	-	Central line-associated bloodstream infections
Öncül 2014	Turkey, burn ICU, all ages	-	-	0%	0%	-	-	-	-	-
Ong 2015	Netherlands, mixed ICUs, adults	-	-	28.57%	0%	-	-	-	-	-
Orsi 2015	Italy, general ICU, adults	-	-	8.7%	0%	-	-	-	-	-
Salmanov 2019b	Ukraine, mixed adults, paediatric and neonatal ICUs	-	-	8.06%	-	-	-	-	-	-
Schwab 2018	Germany, mixed ICUs, all ages	-	-	16.53%	-	-	-	-	-	Primary bloodstream infections
Süner 2015	Turkey, central ICU, adults	-	-	9.38%	-	-	-	-	-	-
Tomaszewski 2019	Poland, mixed ICUs, adults + children	-	-	15%	10%	-	-	-	-	-
Viderman 2018	Kazakkhstan, mixed ICU	-	-	22.64%	-	-	-	-	-	-
Walaszek 2018a	Poland, general adult ICUs	-	-	8.97%	-	-	-	-	-	Primary bloodstream infections
Walaszek 2018b	Poland, general adult ICUs	-	-	7.32% (all HA-BSI); 11.69% (primary HA-BSI); 3.45% (secondary HA-BSI)	0%	-	-	-	-	-
Yetkin 2018	Turkey, mixed adult and paediatric ICUs	-	-	5.37%	-	-	-	-	-	-
Baier 2018	Germany, neonatal ICU	-	-	-	-	8.7%	-	-	-	-
Bolat 2012	Turkey, neonatal ICU	-	-	-	-	1.2%	0%	-	-	-
Crivaro	Italy,	-	-	-	-	2.08%	0%	-	-	Central line-

2015	neonatal ICU									associated bloodstream infections
Djordjevic 2015	Serbia, neonatal ICU	-	-	-	-	0%	0%	-	-	-
Pérerz Lopéz 2013	United Kingdom, neonatal ICU	-	-	-	-	6.06%	-	-	-	-
Verstraete 2014	Belgium, neonatal ICU	-	-	-	-	6.46%	-	-	-	-
Yalaz 2012	Turkey, neonatal ICU	-	-	-	-	11.11%	-	-	-	Catheter-associated bloodstream infections
Gecgel 2016	Turkey, clinical departments and ICUs of cardiology and cardiovascular surgery	-	-	-	-	-	-	12.09%	-	-
Guembe 2017	Spain, internal medicine, adults	-	-	-	-	-	-	5.56%	-	Catheter-associated bloodstream infections
Kołpa 2019	Poland, surgical unit	-	-	-	-	-	-	0%	0%	-
Tsitsopoulos 2016	Greece, neurosurgical unit	-	-	-	-	-	-	12.43%	0%	-

Supplementary Table S7. Vancomycin resistance proportions among *Enterococcus spp.* isolates from patients with HAIs

Study		Hospital wide	ICU	NICU	Other wards	Comment
**All hospital-acquired infections**						
Mancini 2016	Italy, hospital wide	2.59%	-	-	-	-
Ott 2013	Germany, hospital wide	11.54%	-	-	-	-
Salmanov 2019a	Ukraine, hospital wide	11.29%	-	-	-	-
Atici 2016	Turkey, Paediatric ICU	-	37.5%	-	-	-
Boncagni 2015	Italy, mixed medical-surgical ICUs, adults	-	24.14%	-	-	-
Erayman 2016	Turkey, neurosurgical ICU, adults	-	40%	-	-	-
Inan 2012	Turkey, medical-surgical ICU	-	12.5%	-	-	Device-associated infections
Kostakoğlu 2016	Turkey, mixed ICUs, adults	-	5.26%	-	-	-
Öncül 2014	Turkey, burn ICU, all ages	-	0%	-	-	-
Salmanov 2019b	Ukraine, mixed adults, paediatric and neonatal ICUs	-	20%	-	-	-
Viderman 2019	Kazakkhstan, mixed ICU, adults	-	0%	-	-	-
Walaszek 2018a	Poland, general adult ICUs	-	7.41%	-	-	-
Yetkin 2018	Turkey, mixed adult and paediatric ICUs	-	15.38%	-	-	-
Bolat 2012	Turkey, neonatal ICU	-	-	0%	-	-

Crivaro 2015	Italy, neonatal ICU	-	-	0%	-	-
Cura 2016	Turkey, neonatal ICU	-	-	0%	-	-
**Hospital-acquired bloodstream infections**						
Blackburn 2012	United Kingdom, hospital wide	8.94%	-	-	-	-
Blot 2019	Belgium, hospital wide	2.69%	-	-	-	-
Brady 2017	Ireland, hospital wide	33.25%	-	-	-	-
Deptuła 2018	Poland, hospital wide	33.33%	-	-	-	Catheter-associated bloodstream infections
Huttunen 2015	Finland, hospital wide	0%	-	-	-	-
Kontula 2018	Finland, hospital wide	0.5%	-	-	-	-
Pinholt 2014	Denmark, hospital wide	1.68%	-	-	-	-
Sante 2019	Spain, hospital wide	1.12%	-	-	-	Secondary bloodstream infections
Venturini 2016	Italy, hospital wide	0%	-	-	-	Central line-associated bloodstream infections
Atilla 2017	Turkey, mixed ICUs, adults	-	9.52%			Central line-associated bloodstream infections
Culshaw 2014	Italy, mixed ICU, adults	-	20.51% (all HA-BSI); 33.33% (central line-associated BSI)	-	-	-
De Santis 2015	United Kingdom, mixed adult ICU	-	33.33%	-	-	-
Djuric 2019	Serbia, trauma-surgical ICUs, adults	-	66.67%	-	-	-
Kouni 2019	Greece, paediatric and neonatal ICUs, paediatric oncology unit	-	25%	-	-	Central line-associated bloodstream infections
Ong 2015	Netherlands, mixed ICUs, adults	-	0%	-	-	-
Orsi 2015	Italy, general ICU, adults	-	0%	-	-	-
Tomaszewski 2019	Poland, mixed ICUs, adults + children	-	66.67%	-	-	-
Walaszek 2018b	Poland, general adult ICUs	-	0%	-	-	-
Bolat 2012	Turkey, neonatal ICU	-	-	0%	-	-
Crivaro 2015	Italy, neonatal ICU	-	-	0%	-	-
Tsitsopoulos 2016	Greece, neurosurgical unit	-	-	-	0%	-

Supplementary Table S8. Risk of bias assessment of included studies

	External validity				Internal validity					
**Item**	**1**	**2**	**3**	**4**	**5**	**6**	**7**	**8**	**9**	**10**
**Hospital-wide studies**										
Avci 2012	*NA*	high	low	low	low	low	high	low	low	high
Barbato 2019	*NA*	high	low	low	low	low	high	low	low	low
Blackburn 2012	*NA*	high	low	low	low	low	high	low	low	low
Blot 2019	*NA*	high	low	low	low	low	high	low	low	high
Brady 2017	*NA*	high	low	low	low	high	high	low	low	low
Cardoso 2013	*NA*	high	low	low	low	low	high	low	low	high
Ciofi degli Atti 2011	*NA*	high	low	low	low	low	high	low	low	low
Deptuła 2018	*NA*	low	low	low	low	low	high	low	low	low
De Angelis 2018	*NA*	high	low	low	low	high	low	low	low	high
Erdem 2015	*NA*	high	low	low	low	high	high	low	low	high
Green 2015	*NA*	high	low	low	low	high	high	low	low	low
Gubbels 2017	*NA*	low	low	low	low	low	high	low	low	high
Hopmans 2020	*NA*	low	low	low	low	low	high	low	low	low
Huttunen 2015	*NA*	high	low	low	low	low	low	high	low	high
Kolpa 2018b	*NA*	high	low	low	low	low	high	low	low	high
Kontula 2018	*NA*	low	low	low	low	low	high	low	low	high
Mancini 2016	*NA*	high	low	low	low	low	low	low	low	high
Marani 2016	*NA*	high	low	low	low	low	high	low	low	low
Ott 2013	*NA*	high	low	low	low	low	high	low	low	low
Pérerz Lopéz 2013	*NA*	high	low	low	low	low	low	low	low	high
Pinholt 2014	*NA*	high	low	low	low	low	low	low	low	high
Raka 2019	*NA*	low	low	low	low	low	high	low	low	high
Ryan 2015	*NA*	high	low	low	low	low	low	low	low	low
Saliba 2018	*NA*	high	low	low	low	low	low	low	low	high
Salmanov 2019a	*NA*	high	low	low	low	low	low	low	low	low
Sante 2019	*NA*	high	low	low	low	low	high	low	low	high
Venturini 2016	*NA*	high	low	low	low	low	high	low	low	high
Virano 2015	*NA*	high	low	low	low	low	low	low	low	high
**ICU-based studies**										
Atici 2016	*NA*	high	low	low	low	low	low	low	low	high
Atilla 2017	*NA*	high	low	low	low	low	low	low	low	high
Boncagni 2015	*NA*	high	low	low	low	low	high	low	low	low
Bonnet 2019	*NA*	high	low	low	low	low	high	low	low	low
Candevir 2011	*NA*	high	low	low	low	low	low	low	low	high
Celiloğlu 2017	*NA*	high	low	low	low	low	high	low	low	high
Cevik 2011	*NA*	high	low	low	low	low	high	low	low	low
Culshaw 2014	*NA*	high	low	low	low	low	high	low	low	low
Custovic 2014	*NA*	high	low	low	low	low	low	low	low	high
De Santis 2014	*NA*	high	low	low	low	low	high	low	low	high
Deputa 2017	*NA*	low	low	low	low	low	high	low	low	high

Djordjevic 2012	*NA*	high	low	low	low	low	high	low	low	high
Djuric 2019	*NA*	high	low	low	low	low	low	low	low	low
Erayman 2016	*NA*	high	low	low	low	low	high	low	low	high
Geffers 2001	*NA*	high	low	low	low	low	high	low	low	high
Inan 2012	*NA*	high	low	low	low	low	high	low	low	high
Iordanou 2017	*NA*	high	low	low	low	low	high	low	low	low
Kepenekli 2015	*NA*	high	low	low	low	low	high	low	low	high
Kolpa 2018a	*NA*	high	low	low	low	low	high	low	low	low
Kostakoğlu 2016	*NA*	high	low	low	low	low	high	low	low	low
Öncül 2014	*NA*	high	low	low	low	low	low	low	low	high
Ong 2015	*NA*	high	low	low	low	low	high	low	low	low
Orsi 2015	*NA*	high	low	low	low	low	low	high	low	low
Salmanov 2019b	*NA*	high	low	low	low	low	low	low	low	low
Schwab 2018	*NA*	high	low	low	low	low	high	low	low	low
Sutcu 2016	*NA*	high	low	low	low	low	low	low	low	high
Süner 2015	*NA*	high	low	low	low	low	low	low	low	low
Tomaszewski 2019	*NA*	high	low	low	low	low	high	low	low	low
Viderman 2018	*NA*	high	low	low	low	high	low	low	low	low
Viderman 2019	*NA*	high	low	low	low	high	low	low	low	high
Walaszek 2018a	*NA*	high	low	low	low	low	high	low	low	low
Walaszek 2018b	*NA*	high	low	low	low	low	high	low	low	low
Yetkin 2018	*NA*	high	low	low	low	low	low	low	low	high
**Neonatal ICU-based studies**										
Baier 2019	*NA*	high	low	low	low	low	high	low	low	high
Bolat 2012	*NA*	high	low	low	low	low	low	low	low	low
Crivaro 2015	*NA*	high	low	low	low	low	high	low	low	low
Cura 2016	*NA*	high	low	low	low	low	low	low	low	high
Djordjevic 2015	*NA*	high	low	low	low	low	high	low	low	low
Pérerz Lopéz 2013	*NA*	high	low	low	low	low	low	low	low	high
Sadowska-Krawczenko 2012	*NA*	high	low	low	low	low	low	low	low	high
Verstraete 2014	*NA*	high	low	low	low	low	low	low	low	high
Yalaz 2012	*NA*	high	low	low	low	low	high	low	low	low
**Paediatric and neonatal ICU + paediatric oncology unit**										
Kouni 2019	*NA*	high	low	low	low	low	high	low	low	high
**Internal Medicine**										
Guembe 2017	*NA*	high	low	low	low	low	high	low	low	high
**Clinical departments and intensive care units of Cardiology and Cardiovascular surgery**										
Gecgel 2016	*NA*	high	low	low	low	low	low	low	low	high
**Neurosurgery / surgical unit including ICUs**										
Kolpa 2019	*NA*	high	low	low	low	low	high	low	low	high
Tsitsopoulos 2016	*NA*	high	low	low	low	low	low	low	low	low
**Paediatric ward including paediatric ICU**										
Kuzdan 2014	*NA*	high	low	low	low	low	low	low	low	high

Supplementary table S8: Hoy Risk of Bias Assessment

1. Was the study's target population a close representation of the national population in relation to relevant variables?
2. Was the sampling frame a true or close representation of the target population?
3. Was some form of random selection used to select the sample, or was a census undertaken?
4. Was the likelihood of nonresponse bias minimal?
5. Were data collected directly from the subjects (as opposed to a proxy)?
6. Was an acceptable case definition of hospital-acquired infections used in the study?
7. Was the study instrument that measured the parameter of interest to shown to have validity and reliability (microbiological identification and antimicrobial susceptibility testing method and/or interpretation guideline to identify (vancomycin-resistant) enterococci)?
8. Was the same mode of data collection used for all subjects?
9. Was the length of the shortest prevalence period for the parameter of interest appropriate?
10. Were the numerator(s) and denominator(s) for the parameter of interest appropriate*?

* Studies were judged as low risk for item 10 if they only included patients who stayed ≥48h in the hospital since this population represents the population at risk for developing hospital-acquired infections (HAIs). In most epidemiological surveys, infections that occur less than 48h after admission are defined as community-acquired infections and thus, patients with hospital stay less than 48h cannot develop HAIs.

Results for hospital-acquired infections caused by *Enterococcus faecium*

This Supplementary Material describes the results from all included studies that reported data on hospital-acquired infections with *Enterococcus faecium*.

Prevalence of hospital-acquired infections due to (vancomycin-resistant) Enterococcus faecium

For HAIs due to *E. faecium,* four point prevalence studies reported hospital-wide prevalences between 0 and 2.2 cases per 1000 hospital patients, respectively (pooled estimate: 1.6 cases [95% CI 0.91-2.3] per 1000 hospital patients) (Supplementary Table S9). For vancomycin-resistant *E. faecium* Hopmans et al. 2020 (38) reported a hospital-wide point prevalence of 0.08 cases per 1000 hospital patients. For hospital-acquired bloodstream infections, two studies observed point prevalences of 1.3 and 0.35 cases per 1000 hospital patients, respectively.

Supplementary Table S9. Point prevalence of HAIs due to *E. faecium* and vancomycin-resistant *E. faecium* (VREF)

Study		Hospital wide (cases		ICU		NICU	
		E. faecium	VREF	*E. faecium*	VREF	*E. faecium*	VREF
**All Hospital-acquired infections**							
Barbato 2019	Italy, hospital wide	1.25	-	-	-	-	-
Hopmans 2018	Netherlands, hospital wide	2.15	0.08	-	-	-	-
Marani 2016	Italy, hospital wide w/o ICU	1.41	-	-	-	-	-
Raka 2019	Kosovo, hospital wide	0	-	-	-	-	-
**Hospital-acquired bloodstream infections**							
Barbato 2019	Italy, hospital wide	1.25	-	-	-	-	-
Marani 2016	Italy, hospital wide w/o ICU	0.35	-	-	-	-	-
Deptuła 2017	Poland, mixed ICUs, adults	-	-	2.12	-	-	-
Tomaszewski 2019	Poland, mixed ICUs, adults + children	-	-	-	9.76	-	-
**Catheter-associated bloodstream infections**							
Deptuła 2018	Poland, hospital wide	0.06	0.03	-	-	-	-

Incidence of hospital-acquired infections with (vancomycin-resistant) Enterococcus faecium

Two hospital-wide incidence studies showed incidences of 15.3 and 2.4 cases per 1000 patients (Supplementary Table S10). For hospital-acquired bloodstream infections (HA-BSI) due to *E. faecium*, three studies observed incidences between 0.28 and 0.78 cases per 1000 patients, respectively (pooled estimate: 0.58 [95% CI 0.40-0.80]), and 0.35 and 0.06 cases of HA-BSIs due to vancomycin-resistant *E. faecium* per 1000 patients, respectively.

For patients treated in ICUs, six studies reported incidences of HAIs due to *E. faecium* between 0 and 12.5 cases per 1000 ICU patients (pooled estimate: 1.6 [95% CI 0.13-4.21]) (Supplementary Table S10). For HAIs due to vancomycin-resistant *E. faecium*, two of three studies did not find any cases in ICUs, while one study observed an ICU incidence of 4.7 cases per 1000 patients. Two studies with data on HAIs due to *E. faecium* did not find any cases among neonates treated in neonatal ICUs.

Supplementary Table S10. Incidence of HAIs due to *E. faecium* and vancomycin-resistant *E. faecium* (VREF)

Study		Hospital wide (cases per 1000 patients)		ICU (cases per 1000 patients)		NICU (cases per 1000 patients)		Other wards (cases per 1000 patients)	
		E. faecium	VREF	*E. faecium*	VREF	*E. faecium*	VREF	*E. faecium*	VREF
**All Hospital-acquired infections**									
Cardoso 2013	Portugal, hospital wide	2.41	-	-	-	-	-	-	-
Ott 2013	Germany, hospital wide	15.28	-	-	-	-	-	-	-
Atici 2016	Turkey, paediatric ICU	-	-	3.97	-	-	-	-	-
Bonnet 2019	France, mixed ICUs, all ages	-	-	0.58	-	-	-	-	-
Celiloglu 2017	Turkey, paediatric ICU	-	-	0	-	-	-	-	-
Djordjevic 2012	Serbia, neurological ICU, adults	-	-	0	0	-	-	-	-
Öncül 2014	Turkey, burn ICU, all ages	-	-	-	0	-	-	-	-
Salmanov 2019b	Ukraine, mixed adults, paediatric and neonatal ICUs	-	-	12.46	4.67	-	-	-	-
Viderman 2018	Kazakkhstan, mixed ICU	-	-	2.39	-	-	-	-	-
Viderman 2019	Kazakkhstan, mixed ICU, adults	-	-	0	-	-	-	-	-
Crivaro 2015	Italy, neonatal ICU	-	-	-	-	0	0	-	-
Djordjevic 2015	Serbia, neonatal ICU	-	-	-	-	0	0	-	-
Kołpa 2019	Poland, surgical unit	-	-	-	-	-	-	0	0
Kuzdan 2014	Turkey, paediatric ward with ICU	-	-	-	-	-	-	12.56	8.38
**Hospital-acquired bloodstream infections**									

Brady 2017	Ireland, hospital wide	0.78	0.35	-	-	-	-	-	-
De Angelis 2018	Italy, hospital wide	0.55	0.06	-	-	-	-	-	-
Virano 2015	Italy, hospital wide	0.28	-	-	-	-	-	-	-
Celiloğlu 2017	Turkey, paediatric ICU	-	-	0	-	-	-	-	-
Djordjevic 2012	Serbia, neurological ICU, adults	-	-	0	0	-	-	-	-
Öncül 2014	Turkey, burn ICU, all ages	-	-	-	0	-	-	-	-
Ong 2015	Netherlands, mixed ICUs, adults	-	-	-	0	-	-	-	-
Orsi 2015	Italy, general ICU, adults	-	-	3.43	0	-	-	-	-
Viderman 2018	Kazakkhstan, mixed ICU	-	-	1.59	-	-	-	-	-
Baier 2018	Germany, neonatal ICU	-	-	-	-	1.49	-	-	-
Crivaro 2015	Italy, neonatal ICU	-	-	-	-	0	0	-	-
Djordjevic 2015	Serbia, neonatal ICU	-	-	-	-	0	0	-	-
Gecgel 2016	Turkey, clinical departments and ICUs of cardiology and cardiovascular surgery	-	-	-	-	-	-	0.29	-
Kołpa 2019	Poland, surgical unit	-	-	-	-	-	-	0	0
Kuzdan 2014	Turkey, paediatric ward with ICU	-	-	-	-	-	-	2.51	-
Tsitsopoulos 2016	Greece, neurosurgical unit	-	-	-	-	-	-	2.70	0
**Hospital-acquired secondary bloodstream infections**									
Sante 2019	Spain, hospital wide	-	0.01	-	-	-	-	-	-
**Catheter-associated bloodstream infections**									
Candevir 2011	Turkey, mixed adult and paediatric ICUs	-	-	10.83	-	-	-	-	-
Djuric 2019	Serbia, trauma-surgical ICUs, adults	-	-	0	0	-	-	-	-
Guembe 2017	Spain, internal medicine, adults	-	-	-	-	-	-	0	-
**Central line-associated bloodstream infections**									
Crivaro 2015	Italy, neonatal ICU	-	-	-	-	0	0	-	-
**Device-associated infections**									
Iordanou 2017	Cyprus, mixed ICU, adults	-	-	21.66	-	-	-	-	-

Incidence density of hospital-acquired infections with (vancomycin-resistant) Enterococcus faecium

For HA-BSI due to *E. faecium*, three studies reported a hospital-wide incidence density of *E. faecium* between 0 and 0.11 cases per 1000 patient days (pooled estimate: 0.09 [95% CI 0.08-0.11] cases per 1000 patient days) (Supplementary Table S11). For HA-BSI due to vancomycin-resistant *E. faecium* incidence densities between 0 and 0.11 cases per 1000 patient days were observed (pooled estimate: 0.02 [95% CI 0.00-0.08], three studies).

In ICUs, the pooled incidence density of HAIs due to *E. faecium* was 0.14 (95% CI 0.00-0.52) cases per 1000 ICU patient days with an individual study range of 0 to 0.49 cases (Supplementary Table S11). Two out of three studies with data on the ICU incidence density of HAIs due to *E. faecium* did not observe any cases of *E. faecium*, while Salmanov 2019b (66) reported an ICU incidence density of 0.18 cases per 1000 ICU patient days.

With the exception of Pinholt 2014 (82), no study reported population-based data on the population-based incidence or prevalence of HAIs with *E. faecium*. In this large population-based study from Denmark showed that the incidence of monomicrobial HA-BSI with *E. faecium* was 3.5 per 100.000 person-years and 0.05 per 100.000 person-years for monomicrobial HA-BSI with vancomycin-resistant *E. faecium.*

Supplementary Table S11. Incidence density of HAIs due to *E. faecium* and vancomycin-resistant *E. faecium* (VREF)

Study		Hospital wide		ICU		NICU		Other wards	
		E. faecium	**VREF**	***E. faecium***	**VREF**	***E. faecium***	**VREF**	***E. faecium***	**VREF**
**All Hospital-acquired infections**									
Atici 2016	Turkey, paediatric ICU	-	-	0.37 cases per 1000 patient days	-	-	-	-	-
Celiloğlu 2017	Turkey, paediatric ICU	-	-	0 cases per 1000 patient days	-	-	-	-	-
Djordjevic 2012	Serbia, neurological ICU, adults	-	-	0 cases per 1000 patient days	0 cases per 1000 patient days	-	-	-	-
Öncül 2014	Turkey, burn ICU, all ages	-	-	-	0 cases per 1000 patient days	-	-	-	-
Salmanov 2019b	Ukraine, mixed adults, paediatric and neonatal ICUs	-	-	0.49 cases per 1000 patient days	0.18 cases per 1000 patient days	-	-	-	-
Crivaro 2015	Italy, neonatal ICU	-	-	-	-	0 cases per 1000 patient days	0 cases per 1000 patient days	-	-
Djordjevic 2015	Serbia, neonatal ICU	-	-	-	-	0 cases per 1000 patient days	0 cases per 1000	-	-

							patient days		
**Hospital-acquired bloodstream infections**									
De Angelis 2018	Italy, hospital wide	0.11 cases per 1000 patient days	0.01 cases per 1000 patient days	-	-	-	-	-	-
Gubbels 2017	Denmark, hospital wide	0.09 cases per 1000 patient days	-	-	-	-	-	-	-
Huttunen 2015	Finland, hospital wide	-	0 cases per 1000 patient days	-	-	-	-	-	-
Ryan 2015	Ireland, hospital wide	-	0.11 cases per 1000 patient days	-	-	-	-	-	-
Virano 2015	Italy, hospital wide	0.06 cases per 1000 patient days	-	-	-	-	-	-	-
Celiloğlu 2017	Turkey, paediatric ICU	-	-	0 cases per 1000 patient days	-	-	-	-	-
Djordjevic 2012	Serbia, neurological ICU, adults	-	-	0 cases per 1000 patient days	0 cases per 1000 patient days	-	-	-	-
Öncül 2014	Turkey, burn ICU, all ages	-	-	-	0 cases per 1000 patient days	-	-	-	-
Ong 2015	Netherlands, mixed ICUs, adults	-	-	-	0 cases per 1000 patient days	-	-	-	-
Crivaro 2015	Italy, neonatal ICU	-	-	-	-	0.00 cases per 1000 patient days	0 cases per 1000 patient days	-	-
**Hospital-acquired secondary bloodstream infections**									
Sante 2019	Spain, hospital wide	-	0.001 cases per 1000 patient days	-	-	-	-	-	-
**Hospital-acquired catheter-associated bloodstream infections**									
Candevir 2011	Turkey, mixed adult and paediatric ICUs	-	-	1.08 cases per 1000 patient days	-	-	-	-	-
Djuric 2019	Serbia, trauma-surgical ICUs, adults	-	-	0 cases per 1000 patient days	0 cases per 1000 patient days	-	-	-	-
**Central line-associated bloodstream infections**									
Venturini 2016	Italy, hospital wide	-	0 cases per 1000 patient days	-	-	-	-	-	-
Crivaro 2015	Italy, neonatal ICU	-	-	-	-	0 cases per 1000 patient days	0 cases per 1000 patient days	-	-

Mortality

The study Brady et al. 2017 (13) reported an attributable mortality of 18.0% and 19.0% for HA-BSI due to *E. faecium* and vancomycin-resistant *E. faecium* (Supplementary Table S12). Compared to vancomycin-resistant *E. faecium*, slightly a lower mortality was reported for patients with HA-BSI due to vancomycin-sensitive *E. faecium* (17.2 vs. 19.0%). For monomicrobial HA-BSI with *E. faecium* Pinholt et al. 2014 (82) showed a crude mortality of 36.7%.

Supplementary Table S12. Incidence density of HAIs due to *E. faecium*, vancomycin-resistant *E. faecium* (VREF) and vancomycin-sensitive *E. faecium* (VSEF)

**Study**		**Hospital-wide**		
		E. faecium	**VREF**	**VSEF**
**Hospital-acquired bloodstream infections**				
Brady 2017	Ireland, hospital-wide	18.05% (attributable mortality)	19.01% (attributable mortality)	17.24% (attributable mortality)
Pinholt 2014	Denmark, hospital-wide	36.71% (30-day mortality, monomicrobial BSI)	-	-

The proportion of (vancomycin-resistant) Enterococcus faecium among all microorganisms causing HAIs

As reported by six hospital-wide studies, the proportion of *E. faecium* among all microorganisms isolated from HAI patients ranged between 0 and 10.7% (pooled estimate: 3.8% [95%CI 1.9-6.3%]) (Supplementary Table S13). Hopmans et al. (38) showed that 0.19% of all HAI pathogens identified were vancomycin-resistant *E. faecium*. Among pathogens isolated from patients with hospital-acquired bloodstream infections, *E. faecium* accounted for 4.9% (95% CI 0.95-11.2%) of all microorganisms identified (range: 2.4-12.1%, 5 studies). In ICUs, *E. faecium* proportions were generally low (pooled estimate: 0.69% [95% CI 0.11-1.6%], range: 0-3.1%) and three out of seven studies did not identified *E. faecium* in isolates from patients with HAIs (Supplementary Table S13). While two studies did not observe any cases of vancomycin-resistant *E. faecium* among isolated HAI pathogens, Salmanov 2019b (66) reported that vancomycin-resistant *E. faecium* accounted for 0.2% of all HAI pathogens. In isolates from ICU patients with hospital-acquired bloodstream infections, the pooled proportion of *E. faecium* among all HAI pathogens was 3.9% (95% CI 0.92-8.2%, range: 0-10.6%, 6 studies) (Supplementary Table S13).

Supplementary Table S13. Proportion of (vancomycin-resistant) *Enterococcus faecium* among all microorganisms causing HAIs

Study		Hospital wide		ICU		NICU		Other wards		Comment
		E. faecium	**VREF**	***E. faecium***	**VREF**	***E. faecium***	**VREF**	***E. faecium***	**VREF**	
**All Hospital-acquired infections**										
Barbato 2019	Italy, hospital wide	0.8%	-	-	-	-	-	-	-	-
Cardoso 2013	Portugal, hospital wide	3.41%	-	-	-	-	-	-	-	-
Hopmans 2020	Netherlands, hospital wide	5.2%	0.19%	-	-	-	-	-	-	-
Marani 2016	Italy, hospital wide w/o ICU	3.77%	-	-	-	-	-	-	-	-
Ott 2013	Germany, hospital wide	10.74%	-	-	-	-	-	-	-	-
Raka 2019	Kosovo, hospital wide	0%	0%	-	-	-	-	-	-	-
Atici 2016	Turkey, paediatric ICU	-	-	1.84%	-	-	-	-	-	-
Bonnet 2019	France, mixed ICUs, all ages	-	-	0,61%	-	-	-	-	-	-
Celiloğlu 2017	Turkey, paediatric ICU	-	-	0%	0%	-	-	-	-	-
Djordjevic 2012	Serbia, neurological ICU, adults	-	-	0%	0%	-	-	-	-	-
Öncül 2014	Turkey, burn ICU, all ages	-	-	-	0%	-	-	-	-	-
Salmanov 2019b	Ukraine, mixed adults, paediatric and neonatal	-	-	3.05%	1.15%	-	-	-	-	-

	ICUs									
Viderman 2018	Kazakkhstan, mixed ICU	-	-	0.8%	-	-	-	-	-	-
Viderman 2019	Kazakkhstan, mixed ICU, adults	-	-	0%	0%	-	-	-	-	-
Djordjevic 2015	Serbia, neonatal ICU	-	-	-	-	0%	0%	-	-	-
Kołpa 2019	Poland, surgical unit	-	-	-	-	-	-	0%	0%	-
Kuzdan 2014	Turkey, paediatric ward with ICU	-	-	-	-	-	-	6.22%	4.15%	-
**Hospital-acquired bloodstream infections**										
Barbato 2019	Italy, hospital wide	2.38%	-	-	-	-	-	-	-	-
Deptuła 2018	Poland, hospital wide	2.48%	1.24%	-	-	-	-	-	-	Catheter-associated bloodstream infections
Gubbels 2017	Denmark, hospital wide	12.1%	-	-	-	-	-	-	-	-
Huttunen 2015	Finland, hospital wide	-	0%	-	-	-	-	-	-	-
Marani 2016	Italy, hospital wide w/o ICU	4.76%	-	-	-	-	-	-	-	-
Sante 2019	Spain, hospital wide	-	0.19%	-	-	-	-	-	-	Secondary bloodstream infections
Venturini 2016	Italy, hospital wide	0%	0%	-	-	-	-	-	-	Central line-associated bloodstream infections
Virano 2015	Italy, hospital wide	4.08%	-	-	-	-	-	-	-	-
Candevir 2011	Turkey, mixed adult and paediatric ICUs	-	-	10.6%	-	-	-	-	-	Catheter-associated bloodstream infections
Celiloğlu 2017	Turkey, paediatric ICU	-	-	0%	0%	-	-	-	-	-
Deptuła 2017	Poland, mixed ICUs, adults	-	-	3.28%	-	-	-	-	-	-
Djuric 2019	Serbia, trauma-surgical ICUs, adults	-	-	0%	0%	-	-	-	-	Catheter-associated bloodstream infections
Öncül 2014	Turkey, burn ICU, all ages	-	-	-	0%	-	-	-	-	-
Ong 2015	Netherlands, mixed ICUs, adults	-	-	-	0%	-	-	-	-	-
Orsi 2015	Italy, general ICU, adults	-	-	3.48%	0%	-	-	-	-	-
Tomaszewski 2019	Poland, mixed ICUs, adults + children	-	-	-	10%	-	-	-	-	-
Viderman 2018	Kazakkhstan, mixed ICU	-	-	3.77%	-	-	-	-	-	-
Baier 2019	Germany, neonatal ICU	-	-	-	-	4.35%	-	-	-	-
Crivaro 2015	Italy, neonatal ICU	-	-	-	-	0%	0%	-	-	Central line-associated bloodstream infections
Djordjevic 2015	Serbia, neonatal ICU	-	-	-	-	0%	0%	-	-	-
Gecgel 2016	Turkey, clinical departments and ICUs of cardiology and cardiovascular surgery	-	-	-	-	-	-	2.93%	-	-
Guembe 2017	Spain, internal medicine, adults	-	-	-	-	-	-	0%	0%	Central line-associated bloodstream infections
Kołpa 2019	Poland, surgical unit	-	-	-	-	-	-	0%	0%	-
Kuzdan	Turkey,	-	-	-	-	-	-	5.17%	-	-

2014	paediatric ward with ICU									
Tsitsopoulos 2016	Greece, neurosurgical unit	-	-	-	-	-	-	4.23%	0%	-

Vancomycin resistance proportions in Enterococcus faecium isolates from patients with HAIs

As reported by Hopmans et al. 2020 (38), 3.7% of all *E. faecium* isolates from patients with HAIs were vancomycin-resistant (Supplementary Table 14). In ICUs, Salmanov 2019b (66) reported a VREF proportion of 37.5%. Among *E. faecium* isolates from patients with hospital-acquired bloodstream infections, vancomycin resistance proportions ranged between 1.5 and 50.0% (pooled estimate: 18.3% [95%CI 0.5-48.3%], 4 studies).

Supplementary Table S14. Proportion of vancomycin resistance among all *E. faecium* isolates from patient with HAIs

Study		Hospital wide	ICU	Other wards	Comment
		E. faecium	*E. faecium*	*E. faecium*	
**All hospital-acquired infections**					
Hopmans 2020	Netherlands, hospital wide	3.74%	-	-	-
Salmanov 2019b	Ukraine, mixed adults, paediatric and neonatal ICUs	-	37.5%	-	-
Kuzdan 2020	Turkey, paediatric ward with ICU	-	-	66.67%	-
**Hospital-acquired bloodstream infections**					
Brady 2017	Ireland, hospital wide	45.49%	-	-	-
De Angelis 2018	Italy, hospital wide	10.46%	-	-	-
Deptuła 2018	Poland, hospital wide	50%	-	-	Catheter-associated bloodstream infections
Pinholt 2014	Denmark, hospital wide	1.45%	-	-	-
Orsi 2015	Italy, general ICU, adults	-	0%	-	-
Tsitsopoulos 2016	Greece, neurosurgical unit	-	-	0%	-

References of the Supplementary Material

1. Atici S, Soysal A, Kepenekli Kadayifci E, Karaaslan A, Akkoç G, Yakut N, et al. Healthcare-associated infections in a newly opened pediatric intensive care unit in Turkey: Results of four-year surveillance. Journal of infection in developing countries. 2016;10(3):254-9.
2. Garner J, Jarvis W, Emori TG, Horan T, Hughes J. CDC Definitions for Nosocomial Infections. American journal of infection control. 1988;16:128-40.
3. Atilla A, Doğanay Z, Kefeli Çelik H, Demirağ MD, S SK. Central line-associated blood stream infections: characteristics and risk factors for mortality over a 5.5-year period. Turkish journal of medical sciences. 2017;47(2):646-52.
4. Horan TC, Andrus M, Dudeck MA. CDC/NHSN surveillance definition of health care-associated infection and criteria for specific types of infections in the acute care setting. American journal of infection control. 2008;36(5):309-32.
5. Avci M, Ozgenc O, Coskuner SA, Olut AI. Hospital acquired infections (HAI) in the elderly: comparison with the younger patients. Archives of gerontology and geriatrics. 2012;54(1):247-50.
6. Baier C, Pirr S, Ziesing S, Ebadi E, Hansen G, Bohnhorst B, et al. Prospective surveillance of bacterial colonization and primary sepsis: findings of a tertiary neonatal intensive and intermediate care unit. The Journal of hospital infection. 2019;102(3):325-31.
7. Barbato D, Castellani F, Angelozzi A, Isonne C, Baccolini V, Migliara G, et al. Prevalence survey of healthcare-associated infections in a large teaching hospital. Annali di igiene : medicina preventiva e di comunita. 2019;31(5):423-35.
8. Blackburn RM, Henderson KL, Minaji M, Muller-Pebody B, Johnson AP, Sharland M. Exploring the Epidemiology of Hospital-Acquired Bloodstream Infections in Children in England (January 2009-March 2010) by Linkage of National Hospital Admissions and Microbiological Databases. Journal of the Pediatric Infectious Diseases Society. 2012;1(4):284-92.
9. Blot K, Hammami N, Blot S, Vogelaers D, Lambert M-L. Increasing burden of Escherichia coli, Klebsiella pneumoniae, and Enterococcus faecium in hospital-acquired bloodstream infections (2000–2014): A national dynamic cohort study. Infection Control & Hospital Epidemiology. 2019;40(6):705-9.
10. Bolat F, Uslu S, Bolat G, Comert S, Can E, Bulbul A, et al. Healthcare-associated infections in a Neonatal Intensive Care Unit in Turkey. Indian pediatrics. 2012;49(12):951-7.
11. Boncagni F, Francolini R, Nataloni S, Skrami E, Gesuita R, Donati A, et al. Epidemiology and clinical outcome of Healthcare-Associated Infections: a 4-year experience of an Italian ICU. Minerva anestesiologica. 2015;81(7):765-75.
12. Bonnet V, Dupont H, Glorion S, Aupée M, Kipnis E, Gérard JL, et al. Influence of bacterial resistance on mortality in intensive care units: a registry study from 2000 to 2013 (IICU Study). The Journal of hospital infection. 2019;102(3):317-24.
13. Brady M, Oza A, Cunney R, Burns K. Attributable mortality of hospital-acquired bloodstream infections in Ireland. Journal of Hospital Infection. 2017;96(1):35-41.
14. Candevir Ulu A, Kurtaran B, Kibar F, KarakoÇ E, Aksu H, TaŞOva Y. Invasive device-associated nosocomial infections of a teaching hospital in Turkey; four years' experience. Turkish journal of medical sciences. 2011;41:137-47.
15. Cardoso T, Ribeiro O, Aragão I, Costa-Pereira A, Sarmento A. Differences in microbiological profile between community-acquired, healthcare-associated and hospital-acquired infections. Acta medica portuguesa. 2013;26(4):377-84.
16. Yoğun Ç, Ünitesindeki B, Enfeksiyonlarının H, Celiloglu C, Tolunay O, Sucu A, et al. Assessment of Healthcare-Associated Infections in the Pediatric Intensive Care Unit Original Investigation / Özgün Araştırma. 2017.
17. Cevik S, Bosnak V, Namiduru M, Karaoglan I, Mete A. Invasive device-associated hospital infection rates, etiological agents, and their antibiotic susceptibilities in the medical intensive care unit of a university hospital in Turkey. Turkish journal of medical sciences. 2013;43:33-8.

18. Ciofi Degli Atti ML, Cuttini M, Ravà L, Ceradini J, Paolini V, Ciliento G, et al. Trend of healthcare-associated infections in children: annual prevalence surveys in a research hospital in Italy, 2007-2010. The Journal of hospital infection. 2012;80(1):6-12.
19. Crivaro V, Bogdanović L, Bagattini M, Iula VD, Catania M, Raimondi F, et al. Surveillance of healthcare-associated infections in a neonatal intensive care unit in Italy during 2006-2010. BMC infectious diseases. 2015;15:152.
20. Culshaw N, Glover G, Whiteley C, Rowland K, Wyncoll D, Jones A, et al. Healthcare-associated bloodstream infections in critically ill patients: descriptive cross-sectional database study evaluating concordance with clinical site isolates. Annals of intensive care. 2014;4:34.
21. Cura C, Ozen M, Akaslan Kara A, Alkan G, Sesli Cetin E. Health care-associated infection surveillance in a tertiary neonatal intensive care unit: A prospective clinical study after moving to a new building. American journal of infection control. 2016;44(1):80-4.
22. Custovic A, Smajlovic J, Tihic N, Hadzic S, Ahmetagic S, Hadzagic H. Epidemiological monitoring of nosocomial infections caused by acinetobacter baumannii. Medical archives (Sarajevo, Bosnia and Herzegovina). 2014;68(6):402-6.
23. De Angelis G, Fiori B, Menchinelli G, D'Inzeo T, Liotti FM, Morandotti GA, et al. Incidence and antimicrobial resistance trends in bloodstream infections caused by ESKAPE and Escherichia coli at a large teaching hospital in Rome, a 9-year analysis (2007-2015). European journal of clinical microbiology & infectious diseases : official publication of the European Society of Clinical Microbiology. 2018;37(9):1627-36.
24. De Santis V, Gresoiu M, Corona A, Wilson AP, Singer M. Bacteraemia incidence, causative organisms and resistance patterns, antibiotic strategies and outcomes in a single university hospital ICU: continuing improvement between 2000 and 2013. The Journal of antimicrobial chemotherapy. 2015;70(1):273-8.
25. Deptuła A, Trejnowska E, Dubiel G, Żukowski M, Misiewska-Kaczur A, Ozorowski T, et al. Prevalence of healthcare-associated infections in Polish adult intensive care units: summary data from the ECDC European Point Prevalence Survey of Hospital-associated Infections and Antimicrobial Use in Poland 2012-2014. The Journal of hospital infection. 2017;96(2):145-50.
26. European Centre for Disease Prevention and Control. Point prevalence survey of healthcare-associated infections and antimicrobial use in European acute care hospitals - protocol version 4.3. Stockholm: ECDC; 2012.
27. Deptuła A, Trejnowska E, Dubiel G, Wanke-Rytt M, Deptuła M, Hryniewicz W. Healthcare associated bloodstream infections in Polish hospitals: prevalence, epidemiology and microbiology-summary data from the ECDC Point Prevalence Survey of Healthcare Associated Infections 2012-2015. European journal of clinical microbiology & infectious diseases : official publication of the European Society of Clinical Microbiology. 2018;37(3):565-70.
28. Djordjevic Z, Jankovic S, Gajovic O, Djonovic N, Folic N, Bukumiric Z. Hospital infections in a neurological intensive care unit: incidence, causative agents and risk factors. Journal of infection in developing countries. 2012;6(11):798-805.
29. Djordjevic ZM, Markovic-Denic L, Folic MM, Igrutinovic Z, Jankovic SM. Health care–acquired infections in neonatal intensive care units: Risk factors and etiology. American journal of infection control. 2015;43(1):86-8.
30. Olivera D, Ljiljana M-D, Bojan J, Vesna B. High incidence of multiresistant bacterial isolates from bloodstream infections in trauma emergency department and intensive care unit in Serbia. Acta Microbiologica et Immunologica Hungarica AMicr. 2019;66(3):307-25.
31. Erayman I, Erdi M, Kalkan E, Karatas Y, Kaya B, Keskin F, et al. Evaluation of nosocomial infections and related risk factors in a neurosurgery intensive care unit. International Journal of Clinical and Experimental Medicine. 2016;9:7334-8.
32. Erdem D, Akan B, Kanyilmaz D, Demirelli G, Esingen S, Ornek D, et al. The association between total parenteral nutrition and central line-associated bloodstream infection. Acta Medica Mediterranea. 2015;31:1163-7.
33. Karadağ Geçgel S, Demircan N. The epidemiology of pathogen microorganisms in hospital acquired infections. 2016;9:22310-6.

34. Geffers C, Gastmeier P. Nosocomial infections and multidrug-resistant organisms in Germany: epidemiological data from KISS (the Hospital Infection Surveillance System). Deutsches Arzteblatt international. 2011;108(6):87-93.
35. Green N, Johnson AP, Henderson KL, Muller-Pebody B, Thelwall S, Robotham JV, et al. Quantifying the Burden of Hospital-Acquired Bloodstream Infection in Children in England by Estimating Excess Length of Hospital Stay and Mortality Using a Multistate Analysis of Linked, Routinely Collected Data. Journal of the Pediatric Infectious Diseases Society. 2015;4(4):305-12.
36. Gubbels S, Nielsen J, Voldstedlund M, Kristensen B, Schønheyder HC, Ellermann-Eriksen S, et al. National Automated Surveillance of Hospital-Acquired Bacteremia in Denmark Using a Computer Algorithm. Infection control and hospital epidemiology. 2017;38(5):559-66.
37. Guembe M, Pérez-Granda MJ, Capdevila JA, Barberán J, Pinilla B, Martín-Rabadán P, et al. Nationwide study on peripheral-venous-catheter-associated-bloodstream infections in internal medicine departments. The Journal of hospital infection. 2017;97(3):260-6.
38. Hopmans TEM, Smid EA, Wille JC, van der Kooi TII, Koek MBG, Vos MC, et al. Trends in prevalence of healthcare-associated infections and antimicrobial use in hospitals in the Netherlands: 10 years of national point-prevalence surveys. The Journal of hospital infection. 2020;104(2):181-7.
39. Huttunen R, Åttman E, Aittoniemi J, Outinen T, Syrjänen J, Kärki T, et al. Nosocomial bloodstream infections in a Finnish tertiary care hospital: a retrospective cohort study of 2175 episodes during the years 1999–2001 and 2005–2010. Scandinavian Journal of Infectious Diseases. 2014;47.
40. Inan A, Ozgültekin A, Senbayrak S, ozturk engin D, Turan G, Ceran N, et al. Alterations in Bacterial Spectrum and Increasing Resistance Rates in Isolated Microorganisms from Device-Associated Infections in an Intensive Care Unit of a Teaching Hospital in Istanbul (2004–2010). Japanese journal of infectious diseases. 2012;65:146-51.
41. Iordanou S, Middleton N, Papathanassoglou E, Raftopoulos V. Surveillance of device associated infections and mortality in a major intensive care unit in the Republic of Cyprus. BMC infectious diseases. 2017;17(1):607.
42. Kepenekli E, Soysal A, Yalindag-Ozturk N, Ozgur O, Ozcan I, Devrim I, et al. Healthcare-Associated Infections in Pediatric Intensive Care Units in Turkey: a National Point-Prevalence Survey. Jpn J Infect Dis. 2015;68(5):381-6.
43. Kołpa M, Wałaszek M, Gniadek A, Wolak Z, Dobroś W. Incidence, Microbiological Profile and Risk Factors of Healthcare-Associated Infections in Intensive Care Units: A 10 Year Observation in a Provincial Hospital in Southern Poland. Int J Environ Res Public Health. 2018;15(1).
44. European Centre for Disease Prevention and Control. European surveillance of healthcareassociated infections in intensive care units – HAI-Net ICU protocol, version 1.02. Stockholm: ECDC; 2015.
45. Kołpa M, Wałaszek M, Różańska A, Wolak Z, Wójkowska-Mach J. Hospital-Wide Surveillance of Healthcare-Associated Infections as a Source of Information about Specific Hospital Needs. A 5-Year Observation in a Multiprofile Provincial Hospital in the South of Poland. Int J Environ Res Public Health. 2018;15(9).
46. Kołpa M, Wałaszek M, Rózańska A, Wolak Z, wojkowska-Mach J. Epidemiology of Surgical Site Infections and Non-Surgical Infections in Neurosurgical Polish Patients—Substantial Changes in 2003–2017. International Journal of Environmental Research and Public Health. 2019;16:911.
47. Kontula KSK, Skogberg K, Ollgren J, Järvinen A, Lyytikäinen O. The outcome and timing of death of 17,767 nosocomial bloodstream infections in acute care hospitals in Finland during 1999-2014. European journal of clinical microbiology & infectious diseases : official publication of the European Society of Clinical Microbiology. 2018;37(5):945-52.
48. Kostakoglu U, Saylan S, Karataş M, İSkender S, Aksoy F, Yilmaz G. Cost analysis and evaluation of nosocomial infections in intensive care units. Turkish journal of medical sciences. 2016;46:1385-92.
49. Kouni S, Tsolia M, Roilides E, Dimitriou G, Tsiodras S, Skoutelis A, et al. Establishing nationally representative central line-associated bloodstream infection surveillance data for paediatric patients in Greece. The Journal of hospital infection. 2019;101(1):53-9.

50. Kuzdan C, Soysal A, Culha G, Altinkanat G, Soyletir G, Bakir M. Three-year study of health care-associated infections in a Turkish pediatric ward. Journal of infection in developing countries. 2014;8(11):1415-20.
51. Mancini A, Verdini D, La Vigna G, Recanatini C, Lombardi FE, Barocci S. Retrospective analysis of nosocomial infections in an Italian tertiary care hospital. The new microbiologica. 2016;39(3):197-205.
52. Marani A, Napoli C, Berdini S, Montesano M, Ferretti F, Di Ninno F, et al. Point prevalence surveys on healthcare acquired infections in medical and surgical wards of a teaching hospital in Rome. Annali di igiene : medicina preventiva e di comunita. 2016;28(4):274-81.
53. Öncül O, Öksüz S, Acar A, Ülkür E, Turhan V, Uygur F, et al. Nosocomial infection characteristics in a burn intensive care unit: analysis of an eleven-year active surveillance. Burns : journal of the International Society for Burn Injuries. 2014;40(5):835-41.
54. Ong D, Bonten M, Safdari K, Spitoni C, Frencken J, Witteveen E, et al. Epidemiology, Management, and Risk-Adjusted Mortality of ICU-Acquired Enterococcal Bacteremia. Clinical infectious diseases : an official publication of the Infectious Diseases Society of America. 2015;61.
55. Orsi G, Giuliano S, Franchi C, Ciorba V, Protano C, Giordano A, et al. Changed epidemiology of ICU acquired bloodstream infections over 12 years in an Italian teaching hospital. Minerva anestesiologica. 2015;81:980-8.
56. Ott E, Saathoff S, Graf K, Schwab F, Chaberny IF. The prevalence of nosocomial and community acquired infections in a university hospital: an observational study. Deutsches Arzteblatt international. 2013;110(31-32):533-40.
57. Pérerz Lopéz A, Ladhani SN, Breathnach A, Planche T, Heath PT, Sharland M. Trends in paediatric nosocomial bacteraemia in a London tertiary hospital. Acta paediatrica (Oslo, Norway : 1992). 2013;102(10):1005-9.
58. Pinholt M, Ostergaard C, Arpi M, Bruun NE, Schønheyder HC, Gradel KO, et al. Incidence, clinical characteristics and 30-day mortality of enterococcal bacteraemia in Denmark 2006-2009: a population-based cohort study. Clinical microbiology and infection : the official publication of the European Society of Clinical Microbiology and Infectious Diseases. 2014;20(2):145-51.
59. Raka L, Spahija G, Gashi-Gecaj A, Hamza A, Haxhiu E, Rashiti A, et al. Point prevalence survey of healthcare-associated infections and antimicrobial use in Kosovo hospitals. Infectious disease reports. 2019;11(1):7975.
60. Ryan L, O'Mahony E, Wrenn C, Fitzgerald S, Fox U, Boyle B, et al. Epidemiology and molecular typing of VRE bloodstream isolates in an Irish tertiary care hospital. The Journal of antimicrobial chemotherapy. 2015;70.
61. European Centre for Disease Prevention and Control. Point prevalence survey of healthcareassociated infections and antimicrobial use in European acute care hospitals. Stockholm: ECDC; 2013.
62. Sadowska-Krawczenko I, Jankowska A, Kurylak A. Healthcare-associated infections in a neonatal intensive care unit. Arch Med Sci. 2012;8(5):854-8.
63. Saliba P, Hornero A, Cuervo G, Grau I, Jimenez E, García D, et al. Mortality risk factors among non-ICU patients with nosocomial vascular catheter-related bloodstream infections: a prospective cohort study. The Journal of hospital infection. 2018;99(1):48-54.
64. Mermel LA, Allon M, Bouza E, Craven DE, Flynn P, O'Grady NP, et al. Clinical Practice Guidelines for the Diagnosis and Management of Intravascular Catheter-Related Infection: 2009 Update by the Infectious Diseases Society of America. Clinical Infectious Diseases. 2009;49(1):1-45.
65. Salmanov AG, Vdovychenko SY, Litus OI, Litus VI, Bisyuk YA, Bondarenko TM, et al. Prevalence of health care-associated infections and antimicrobial resistance of the responsible pathogens in Ukraine: Results of a multicenter study (2014-2016). American journal of infection control. 2019;47(6):e15-e20.
66. Salmanov A, Litus V, Vdovychenko S, Litus O, Davtian L, Ubogov S, et al. Healthcare-associated infections in intensive care units. Wiadomosci lekarskie (Warsaw, Poland : 1960). 2019;72(5 cz 2):963-9.

67. Sante L, Aguirre-Jaime A, Miguel MA, Ramos MJ, Pedroso Y, Lecuona M. Epidemiological study of secondary bloodstream infections: The forgotten issue. Journal of infection and public health. 2019;12(1):37-42.
68. Schwab F, Geffers C, Behnke M, Gastmeier P. ICU mortality following ICU-acquired primary bloodstream infections according to the type of pathogen: a prospective cohort study in 937 Germany ICUs (2006-2015). PloS one. 2018;13(3):e0194210.
69. Süner A, Karaoğlan I, Mete AO, Namiduru M, Boşnak V, Baydar I. Assessment of bloodstream infections and risk factors in an intensive care unit. Turkish journal of medical sciences. 2015;45(6):1243-50.
70. Sutcu M, Akturk H, Acar M, Salman N, Aydın D, Akgun Karapınar B, et al. Impact of vancomycin-resistant enterococci colonization in critically ill pediatric patients. American journal of infection control. 2016;44(5):515-9.
71. Tomaszewski D, Rybicki Z, Duszyńska W. The Polish Prevalence of Infection in Intensive Care (PPIC): A one-day point prevalence multicenter study. Advances in clinical and experimental medicine : official organ Wroclaw Medical University. 2019;28(7):907-12.
72. Tsitsopoulos P, Iosifidis E, Antachopoulos C, Anestis D, Karantani E, Karyoti A, et al. Nosocomial bloodstream infections in neurosurgery: a 10-year analysis in a center with high antimicrobial drug-resistance prevalence. Acta Neurochirurgica. 2016;158.
73. Venturini E, Montagnani C, Benni A, Becciani S, Biermann KP, De Masi S, et al. Central-line associated bloodstream infections in a tertiary care children's University hospital: a prospective study. BMC infectious diseases. 2016;16(1):725-.
74. Verstraete E, Boelens J, De Coen K, Claeys G, Vogelaers D, Vanhaesebrouck P, et al. Healthcare-associated bloodstream infections in a neonatal intensive care unit over a 20-year period (1992-2011): trends in incidence, pathogens, and mortality. Infection control and hospital epidemiology. 2014;35(5):511-8.
75. Viderman D, Khamzina Y, Kaligozhin Z, Khudaibergenova M, Zhumadilov A, Crape B, et al. An observational case study of hospital associated infections in a critical care unit in Astana, Kazakhstan. Antimicrobial resistance and infection control. 2018;7:57.
76. Viderman D, Brotfain E, Khamzina Y, Kapanova G, Zhumadilov A, Poddighe D. Bacterial resistance in the intensive care unit of developing countries: Report from a tertiary hospital in Kazakhstan. Journal of global antimicrobial resistance. 2019;17:35-8.
77. Virano S, Scolfaro C, Garazzino S, De Intinis C, Ghisetti V, Raffaldi I, et al. Medical care related laboratory-confirmed bloodstream infections in paediatrics. Le infezioni in medicina. 2015;23(2):117-24.
78. Walaszek M, Rozanska A, Bulanda M, Wojkowska-Mach J, Team P. Epidemiology of healthcare-associated infections in Polish intensive care. A multicenter study based on active surveillance. Biomedical papers of the Medical Faculty of the University Palacky, Olomouc, Czechoslovakia. 2018;162(3):190-7.
79. Wałaszek M, Różańska A, Bulanda M, Wojkowska-Mach J, Polish Society of Hospital Infections T. Alarming results of nosocomial bloodstream infections surveillance in Polish intensive care units. Przeglad epidemiologiczny. 2018;72(1):33-44.
80. Yalaz M, Koroglu O, Ulusoy B, Yildiz Atikan B, Akisu M, Vardar F, et al. Evaluation of device-associated infections in a neonatal intensive care unit. The Turkish journal of pediatrics. 2012;54:128-35.
81. Yetkin F, Yakupogullari Y, Kuzucu C, Ersoy Y, Otlu B, Colak C, et al. Pathogens of Intensive Care Unit-Acquired Infections and Their Antimicrobial Resistance: A 9-Year Analysis of Data from a University Hospital. Jundishapur J Microbiol. 2018;11(10):e67716.
82. Pinholt M, Østergaard C, Arpi M, Bruun NE, Schønheyder HC, Gradel KO, et al. Incidence, clinical characteristics and 30-day mortality of enterococcal bacteraemia in Denmark 2006–2009: a population-based cohort study. Clinical Microbiology and Infection. 2014;20(2):145-51.
